# Advances in adhesive hydrogels applied for ophthalmology: An overview focused on the treatment

**DOI:** 10.7150/thno.103266

**Published:** 2025-01-01

**Authors:** Ke Yan, Qinghe Zhang, Qiuping Liu, Yi Han, Zuguo Liu

**Affiliations:** 1 Department of Ophthalmology, The First Affiliated Hospital of University of South China, Hengyang Medical School, University of South China, Hengyang Hunan 421001, China.; 2 Xiamen University affiliated Xiamen Eye Center, Fujian Provincial Key Laboratory of Ophthalmology and Visual Science, Fujian Engineering and Research Center of Eye Regenerative Medicine, Eye Institute of Xiamen University, School of Medicine, Xiamen University, Xiamen Fujian 361005, China.

**Keywords:** adhesive hydrogels, ophthalmology, polymerization and adhesion mechanisms, treatment of ocular diseases

## Abstract

Adhesive hydrogels, composed of hydrophilic polymers arranged in a three-dimensional network, have emerged as a pivotal innovation in ophthalmology due to their ability to securely adhere to ocular tissues while providing sustained therapeutic effects. The eye, with its delicate structure and specific needs, presents unique challenges for drug delivery and tissue regeneration. This review explores the transformative potential of adhesive hydrogels in addressing these challenges across a range of ocular conditions, including corneal injuries, cataracts, glaucoma, vitreoretinal disorders, and ocular trauma. By detailing the mechanisms of polymerization and adhesion, this paper highlights how these materials can be customized for specific ophthalmic applications, offering insights into their current use and future possibilities. The emphasis is placed on the clinical significance and future directions of adhesive hydrogels in advancing ophthalmic therapy, potentially revolutionizing the treatment of complex eye diseases.

## 1. Introduction

The eye is a vital organ and the sole access to vision. Numerous ocular diseases, including corneal and conjunctival trauma, chemical damage, ocular surface diseases such as dry eye 1, 2, refractive errors 3, 4, cataracts 5, 6, glaucoma 7, 8, diabetic retinopathy 9, 10, and retinal detachment 11, 12, affect vision quality to varying degrees. The quality of vision is closely related to a person's quality of life. The World Health Organization (WHO) reported that globally, at least 2.2 billion people suffer from impaired vision at near or far distances 13. At least 1 billion of these cases could have been prevented or are yet to be addressed. Visual impairment also imposes an enormous economic burden, with an estimated annual global productivity loss of $411 billion 13.

Currently, ophthalmic treatments are primarily categorized into medications and surgery. For the anterior segment of the eye, medications are mainly administered as eye drops. However, the efficacy of eye drops is limited by their short duration of action, due to the physical flushing action of tears and blinking, which necessitates frequent applications or higher drug concentrations to achieve therapeutic levels. This often results in poor patient compliance and inconsistent medication adherence. For the posterior segment of the eye, treatments typically involve invasive vitreous injections, requiring multiple doses to compensate for rapid drug metabolism. This approach is associated with a high risk of ocular complications and potential retinal toxicity 14, 15. On the other hand, the surgical treatment of refractive errors, cataracts, glaucoma, and retinal detachment essentially involves ocular wound repair, including the healing of corneal, conjunctival, scleral and retinal wounds. Tissue wound repair requires a certain amount of time, and when patient compliance is poor, it is prone to wound dehiscence, secondary scarring, inflammation, and rejection due to the presence of suture nodules 16-18.

Hydrogels are hydrophilic molecules with three-dimensional (3D) networks that absorb and retain large amounts of water 19. In the last decade, hydrogels have been widely used for ocular drug delivery and wound repair, with several products already on the market, such as ReSure® 20, DEXTENZA® 21. Notably, hydrogels with strong adhesive properties have been developed for drug delivery and ocular tissue repair, offering new methods and ideas for clinical ocular therapy. Since ophthalmic tailored materials must ensure both the structural integrity of the eye and its refractive function, adhesive hydrogels for ocular applications need to possess the following characteristics: 1) excellent biocompatibility and non-toxicity; 2) a suitable surface microstructure to promote cell proliferation and migration; 3) strong adhesion in humid environments; 4) good light transmission; 5) adequate mechanical strength; 6) high stability against significant changes in the ocular environment; 7) simultaneous degradation during the healing process or easy removal after repair is complete; and 8) easy handling.

**Figure 1:**
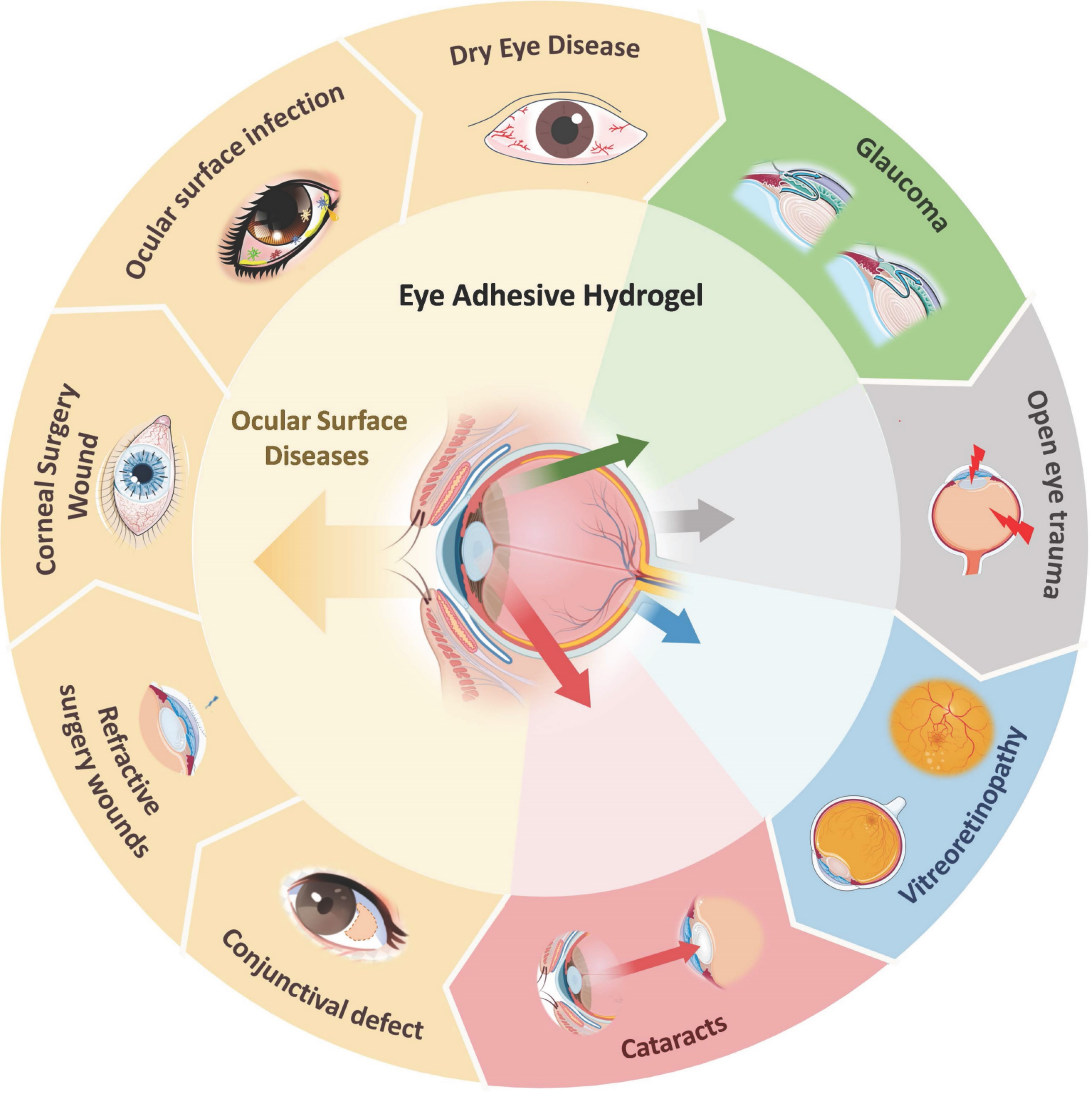
Classification of adhesive hydrogels for ocular applications.

With advances in materials science, researchers have developed various multifunctional ocular adhesive hydrogels that address the challenges mentioned above. However, a comprehensive overview of their design and applications has been lacking. In this review, we present, for the first time, an in-depth analysis of the latest developments in adhesive hydrogels for various ocular tissues. We explore the polymerization and adhesion mechanisms of the currently studied adhesive hydrogels. The main section of the review introduces bioadhesive hydrogel materials specifically designed for different ocular diseases (**Figure 1**) and critically examines their advantages and limitations. Additionally, we summarize current research trends and offer insights into the future design and application of ocular adhesive hydrogels. The aim of this paper is to enhance the understanding of adhesive hydrogels for various ocular tissues, guide the improved design of these materials for diverse ocular applications, and stimulate progress in the development of ocular hydrogels.

## 2. Overview of adhesive hydrogel

**Figure 2:**
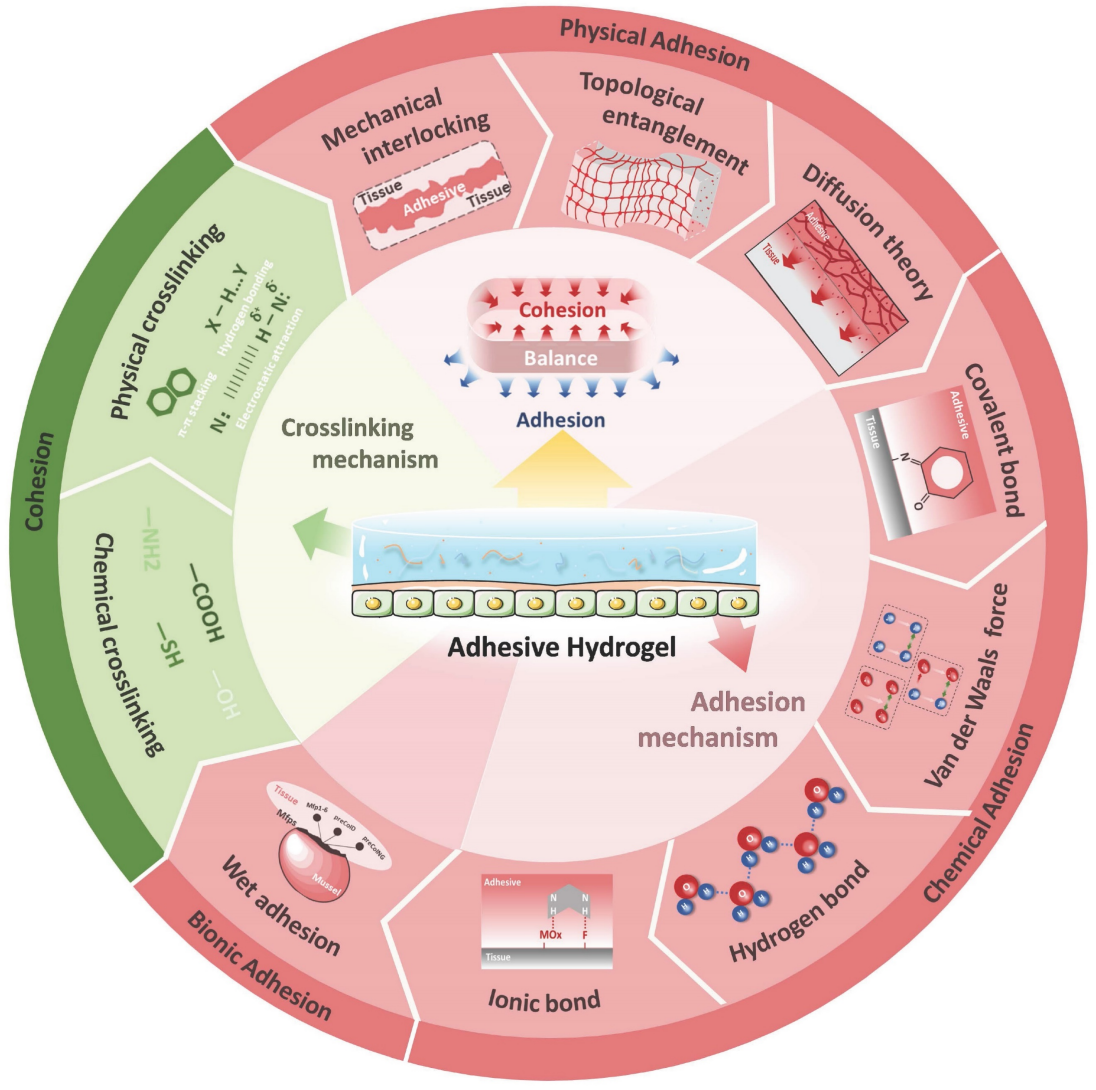
Crosslinking and adhesion mechanisms of adhesive hydrogels.

With the vigorous development of research, hydrogels have gradually evolved from a single network structure to a network structure with multiple interactions. Adhesive hydrogels have also evolved from basic adhesion to multifunctional adhesion with intelligent response. As a special class of hydrogels, this part will briefly describe the crosslinking mechanism of ocular adhesive hydrogels, focusing on the adhesion mechanism (**Figure 2**).

### 2.1 Crosslinking mechanisms for hydrogel

#### 2.1.1 Physical crosslinking

The physical cross-linking method primarily involves physical effects (such as temperature, pressure, electric field, *etc.*) to induce polymer chain cross-linking, forming a network structure. This method typically does not require the addition of chemical crosslinking agents, making the preparation process relatively simple and environmentally friendly. However, most physical cross-linking is reversible and mechanically unstable.

Some polymer hydrogels undergo sol-gel transition due to changes in temperature affecting the entanglement of polymer chains, which is known as temperature-sensitive hydrogel 22. The cornea, conjunctiva, and eyelid tissues, due to their exposure to the external environment, generally maintain a temperature of 32-34°C under normal conditions 23. Commonly used temperature-sensitive polymers include poly N-isopropyl acrylamide (pNIPAAm) 24, poloxamer 25, chitosan 26 and Pluronic F127 27, all of which can form gels at 37°C. Therefore, these materials can remain in a liquid state at room temperature and undergo a sol-gel transition upon contact with the eye surface, enhancing their adhesive properties.

#### 2.1.2 Chemical crosslinking

Chemical crosslinking involves the formation of polymers through chemical reactions between various functional groups. Compared to hydrogels synthesized by physical crosslinking, those synthesized by chemical crosslinking methods exhibit better mechanical strength, tissue adhesion, and stability 28. The commonly used cross-linkable groups include amino (-NH2), carboxyl (-COOH), sulfhydryl (-SH), aldehyde (-CHO) and hydroxyl (-OH). Common chemical crosslinking methods include aldimine condensation, the glutaraldehyde method, and the carbodiimide method. The gel structure formed by chemical crosslinking is generally irreversible unless the chemical bonds are broken. Therefore, this crosslinking method is more advantageous for designing robust adhesive hydrogels and hydrogel patches for ophthalmic applications.

### 2.2 Adhesion mechanism of hydrogel

The adhesive strength of hydrogels is influenced by two crucial factors: cohesion and adhesion. Cohesion refers to the internal force that binds the hydrogel network together, while adhesion measures the strength of the bond between the hydrogel adhesive and the surface of the target tissue 29. Failures in adhesive hydrogels can often be traced back to cohesive failure caused by the rupture of interfacial adhesion or insufficient mechanical stability. Different adhesion mechanisms are suited to different application scenarios, and achieving a balance between cohesion and adhesion is critical. For the complex and tightly structured organ like the eye, hydrogels should be designed by combining various adhesion mechanisms according to the specific characteristics of the adhesion interface. The adhesion mechanisms of hydrogels are diverse and can generally be categorized into physical adhesion (mechanical interlocking, diffusion theory, topological entanglement), chemical adhesion (van der Waals forces, hydrogen bonds, ionic bonds and covalent bonds) and bionic adhesion (catechols, dopamine, and other wet adhesion mechanisms).

#### 2.2.1 Physical adhesion

Mechanical interlocking is a traditional hydrogel adhesion strategy, first discovered on wood by McBain and Hopkins in 1925 30. Mechanical interlocking refers to the interlocking between the adhesive and the microrough structure on the surface of the bonded object 31. According to diffusion theory, adhesion is established through the intermixing (or mutual diffusion) of molecules between the substrate and the adhesive to achieve mutual adhesion. Hydrogels can achieve interdiffusion between polymers through an interpenetrating network structure, enhancing their adhesion properties. Topological entanglement is an emerging approach for hydrogels with prefabricated networks, where the bonding polymer chains act as sutures to bind the adherends through topological connections.

Given the complex structure of ocular tissues, the application of physical adhesion requires consideration of the physical properties and structure of both the polymer and the adhesion interface. Healthy ocular tissues are smooth and tightly bound with a tear film, whereas in pathological states, the cornea and conjunctiva may become rough and edematous, making the tissue structure looser and the tear film uneven. Thus, polymer hydrogels can adaptively fill rough adhesion interfaces through mechanical interlocking and topological entanglement, promoting diffusion within loose tissue structures to increase adhesion, thereby achieving seamless repair or extending drug delivery time.

#### 2.2.2 Chemical adhesion

At the molecular level, chemical adhesion arises from interactions between molecules, such as hydrogen bonds, van der Waals forces, ionic bonds and covalent bonds. Van der Waals forces, also known as intermolecular forces, exist in all polar or nonpolar molecules. These forces are generated by the temporary dipole moments produced by the instantaneous positions of electrons in the molecule. Van der Waals forces are particularly diminished in water, making them less significant for the adhesive properties of hydrogels. However, in ocular applications where external tissues are damaged, van der Waals forces can facilitate reversible adhesion to prevent fluid loss and provide a foundation for subsequent wound treatment.

Hydrogen bonds, stronger than van der Waals forces, involve interactions between a hydrogen atom and an electronegative atom like oxygen or nitrogen 32. Since the eye is a highly hydrated organ, hydrogen bonding plays a crucial role in enhancing adhesion between hydrogels and moist or biological tissue surfaces, making them ideal for eye applications. Additionally, the reversibility of hydrogen bonds balances adhesion with flexibility, adapting hydrogels to ocular movement (blinking and rotation).

Ionic bonds, formed when atoms or chemical groups gain or lose electrons, typically form stronger bonds in moist environments and can break and reform under certain conditions, providing self-healing properties. This reversibility is especially useful for medical hydrogels in prolonged tissue contact, such as those used in the eye.

Covalent bonds, which form when atoms share electrons, create strong, stable connections between molecules 33. Static covalent bonds, such as carbon, amide, siloxane, and carbon-nitrogen bonds, are suitable for applications requiring long-term adhesion (retinal tear repair and biosensors in the fundus). In contrast, dynamic covalent bonds, which can reversibly dissociate and reconfigure in response to environmental changes (pH, heat, light or redox), provide self-healing properties, making them valuable for smart hydrogels and drug delivery systems within the eye 34.

#### 2.2.3 Bionic adhesion

Bionic adhesion mechanisms, often referred to as bio-inspired adhesion, are exemplified by various organisms that exhibit wet adhesion, including marine organisms (mussels, abalone) and amphibians (salamanders, tree frogs). These organisms achieve strong adhesion through chemical reactions or unique microstructures. Inspired by the remarkable adhesion properties of mussel foot proteins, the catechol fraction (dopamine) derived from mussels has gained significant attention 35, 36. Most biological tissues have amino groups on their surfaces, and the eye is no exception. The hydroxyl structure of dopamine can form hydrogen bonds with amino groups at the tissue interface to enhance adhesion. Under certain conditions, DOPA's catechol structure can also form π-cation interactions and covalent bonds, enabling long-lasting adhesion. This capability has been applied in the development of hydrogels for ocular applications, such as retinal hole repair and bio-sensors.

Barnacles and mollusks, which can adhere in moist and variable marine environments, secrete adhesive proteins and polysaccharides, which can be integrated into hydrogel systems to create strong adhesives for treating ocular injuries, modifying the surfaces of ocular implants, and more.

In summary, the design and development of adhesive hydrogels necessitate a thorough consideration of the polymer network type, cross-linking mode, and adhesion mechanism. By rationally combining these factors, hydrogels can be tailored to meet the specific needs of their intended applications, particularly in the complex and dynamic environment of the eye.

## 3. Application of adhesive hydrogel in treatment of ocular disease

The eye is a remarkably intricate and delicate closed cavity structure. Anatomically, the eyeball is segmented into two distinct parts: the anterior segment and the posterior segment. The anterior segment encompasses the cornea, conjunctiva, aqueous humor, iris, and lens, whereas the posterior segment comprises the retina, choroid, sclera, optic nerve, and vitreous humor 37. While each segment exhibits a unique organizational structure, both play a pivotal role in the development and maintenance of visual quality within the eye 38.

Adhesive hydrogel systems tailored to meet the demands of various diseases exhibit diverse functionalities and properties. For instance, in cases of dry eye, glaucoma, and ocular infectious diseases, hydrogels are designed to provide localized adhesion, extending the drug's efficacy and reducing the need for frequent administrations, thus enhancing patient compliance 39-41. For corneal transplantation surgeries, pterygium excision, conjunctival mass removal, and cataract lens replacement, hydrogels focus on sealing wounds and promoting tissue repair 42, 43. In the context of vitreoretinal diseases, hydrogels must not only facilitate the delivery of biologically active ocular substances but also ensure stability and adhesion within the retina 44-46. Bearing these considerations in mind, we will delve into the adhesive hydrogel systems that have been explored based on the classification of ocular diseases.

### 3.1 Ocular surface diseases

Ocular surface diseases refer to diseases that impair the normal structure and function of the corneal conjunctival ocular surface. Here we will detail the research on adhesive hydrogels for the treatment of the corresponding diseases in five areas: dry eye, ocular surface infections, corneal wound surgery, refractive surgery wounds, and conjunctival defects (**Table 1**).

#### 3.1.1 Dry eye

Dry eye disease (DED), a prevalent ocular condition, manifests as an alteration in the quality and quantity of tears stemming from diverse causes 47. Traditional clinical medications for DED primarily consist of eye drops, which are prone to the mechanical movements of tears and eyelids, necessitating frequent administration 48. Consequently, current research endeavors exploring adhesive hydrogels for DED have centered on extending the duration of drug efficacy on the ocular surface, aiming to enhance the treatment of this condition 49, 50.

**Figure 3:**
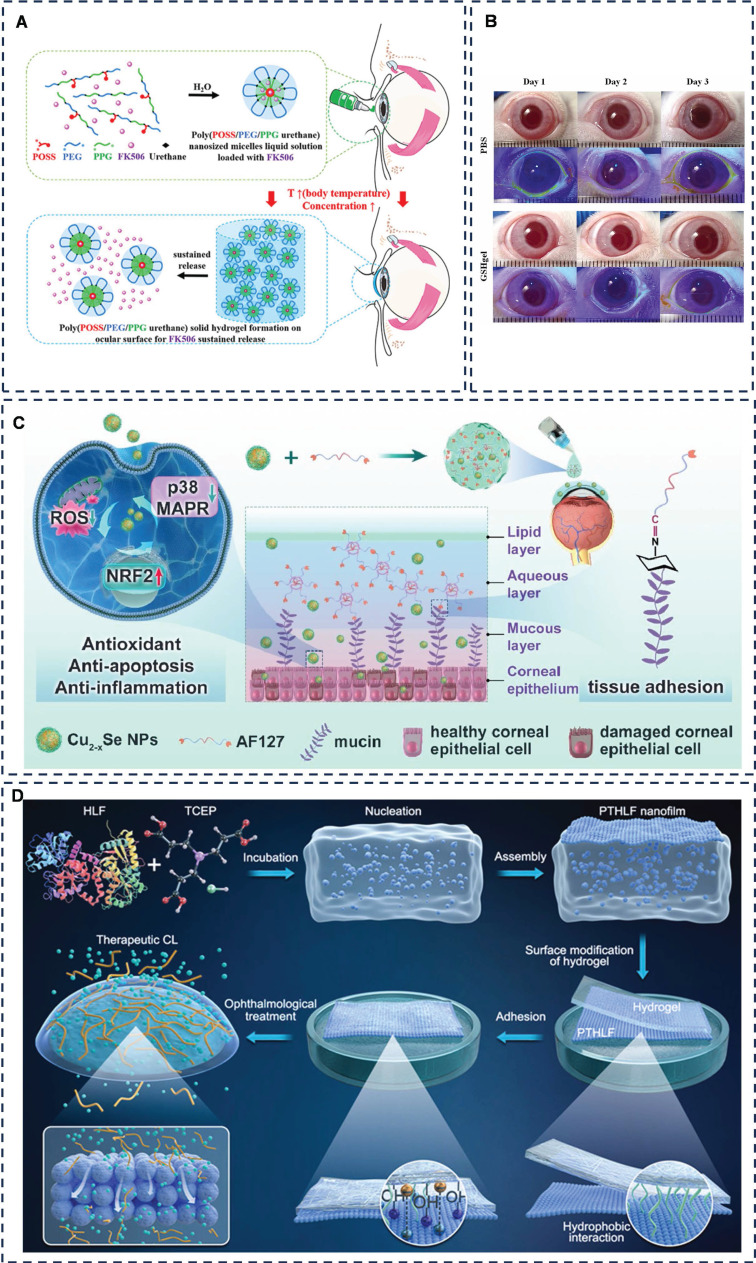
**Representative diagram of adhesive hydrogels applied for dry eye disease. A)** Effectiveness of an ocular adhesive polyhedral oligomeric silsesquioxane hybrid thermo-responsive FK506 hydrogel in a murine model of dry eye. Adapted with permission from 51, Copyright 2022 Elsevier. **B)** Instant Adhesion of Amyloid-like Nanofilms with Wet Surfaces. Adapted with permission from 52, Copyright 2022 American Chemical Society. **C)** A tissue-adhesive F127 hydrogel delivers antioxidative copper-selenide nanoparticles for the treatment of dry eye disease. Adapted with permission from 53, Copyright 2024 Elsevier.** D)** Mucoadhesive phenylboronic acid-grafted carboxymethyl cellulose hydrogels containing glutathione for treatment of corneal epithelial cells exposed to benzalkonium chloride. Adapted with permission from 61, Copyright 2024 Elsevier.

Han *et al.* successfully formulated a thermoresponsive hydrogel loaded with tacrolimus (FK506), utilizing monofunctional polyhedral oligomeric sesquicarbophilic siloxanes (POSS), polyethylene glycol (PEG), polypropylene glycol (PPG) and polyurethane (MPEP) as its constituents (**Figure 3A**) 51. Their results indicated that the MPOSS-PEG-PPG-FK506 (MPEP-FK506) hydrogel exhibited a significantly superior therapeutic effect on dry eye disease (DED) compared to commercial FK506 and PEG-PPG-FK506 (F127-FK506) hydrogels. Notably, the MPEP-FK506 hydrogel adhered uniformly to the cornea even after mice blinked. Furthermore, surface plasmon resonance (SPR) analysis revealed that the hydrophobic POSS groups within the copolymer interact strongly with the exposed hydrophobic domains surrounding the mucin in the cornea, contributing to the hydrogel's adhesion and extending the duration of drug action. This study not only improved the solubility of FK506 but also prolonged its therapeutic effect and enhanced drug utilization, thanks to the strong affinity between the POSS moiety and mucin. Future research could explore additional adhesion mechanisms of this polymer and determine if the POSS moiety possesses a similarly robust affinity for other proteins or tissues.

Drawing inspiration from barnacles' use of the amyloid system to create stable aqueous adhesive surfaces on solids, Qin *et al.* crafted a functional therapeutic contact lens coated with human lactoferrin (HLF) nanomembranes that encapsulate cyclosporine A (CsA) (**Figure 3B**) 52. This innovative design allows for controlled release of CsA only upon application to the eye, resulting in a remarkable 82% increase in bioavailability compared to commercial CsA Restasis®. This study not only introduces a novel therapeutic method for ophthalmic drug delivery, but also holds the potential to accelerate research into biocompatible, wearable contact lens devices. Should the retention of the nanomembrane be detectable post-removal, such devices would offer a more economical solution for clinical applications.

Additionally, Ou *et al.* developed aldehyde-functionalized F127 (AF127) hydrogel eye drops containing multifunctional antioxidant Cu^2^-x selenium nanoparticles (Cu^2^-x Se NPs) to treat dry eye disease (DED) (**Figure 3C**) 53. The investigation revealed that the Cu^2^-x Se NPs @AF127 aqueous gel eye drops adhered well to the ocular surface due to the formation of Schiff base bonds. Compared to F127, the AF127 group exhibited minimal loss of adhesion even after 60 minutes of dispensing, indicating superior adhesion and extended treatment time of the aqueous gel. However, since the study was conducted on anesthetized mice with resting eye muscles, a dynamic assessment of the adhesion effect is necessary. Using larger animals or isolated porcine eyes to mimic human ocular movement would provide a more accurate simulation.

Carboxymethylcellulose (CMC) is one of U.S. Food and Drug Administration (FDA)-approved gel formulations for ophthalmic drug delivery 54. CMC exhibits thermosensitive properties. Lin *et al.* utilized CMC hydrogel by injecting it through the punctum into the lacrimal duct. The CMC undergoes a sol-gel transition in response to temperature changes, leading to increased viscosity and enhanced cohesion, allowing it to settle within the lacrimal duct 55. This adaptation reduces tear drainage, making it a promising approach for managing dry eye disease. Moreover, CMC is inherently viscous and binds to fibronectin and collagen to promote adhesion of corneal epithelial cells 56, 57. Phenylboronic acid (PBA) has two hydroxyl groups on the boron atom and can form high-affinity complexes with the 1.2-cis-diol moiety of sialic acid in mucins, and PBA nanoparticles bind to mucins 4.7-9.1 times more than other mucosal adhesion nanoparticles, such as chitosan-based and sulfate nanoparticles 58, 59. Combining these two points, Cheng* et al.* designed a glutathione (GSH)-rich PBA-grafted CMC hydrogel (PBA-CMC-GSH) (**Figure 3D**) 60. The team demonstrated that the PBA-CMC-GSH hydrogel was effective in alleviating benzalkonium chloride (BAC)-induced apoptosis of corneal epithelial cells and exhibited good biocompatibility without significant eye irritation 61. BAK can induced dry eye-like changes. However, the therapeutic effect of this study was limited to cellular experiments, and further *in vivo* animal experiments must be carried out to validate its therapeutic potential for dry eye disease (DED) by adding a more classical dry eye animal model for further *in vivo* animal experiments. Additionally, studies have explored the use of nano-hydrogels for drug delivery in the treatment of dry eye disease, often utilizing temperature-induced sol-gel transitions to enhance adhesion effects 62, 63.

DED is a complex, multifaceted disease. With advancing knowledge about its underlying mechanisms, the pathogenesis of DED has become increasingly clear. Consequently, the prospect of utilizing ocular surface adhesive hydrogels for DED treatment is highly promising, yet this area of research remains relatively underexplored, particularly in terms of drug delivery. Given that hydrogels are inherently water-retaining materials, they offer inherent advantages in treating dry eye conditions. Thus, we should focus on developing hydrogels loaded with suitable drugs or designing polymer systems that can inhibit inflammation and oxidative stress, tailored specifically to the pathogenesis of dry eye. This approach, which is grounded in maintaining the aqueous balance of the ocular surface, will significantly contribute to more precise and effective treatments for dry eye.

#### 3.1.2 Ocular surface infection

Ocular surface infections are broadly classified into bacterial, viral, fungal, and other microbial types 64. Currently, the treatment of these infections almost exclusively relies on topical medications 65. However, the application of these topical drugs is hampered by challenges such as poor solubility, limited bioavailability, and significant drug loss, necessitating frequent dosing and often leading to suboptimal patient compliance 66, 67. Consequently, there is a pressing need for effective drug delivery methods that can enhance local drug penetration and extend the duration of therapeutic action.

**Figure 4:**
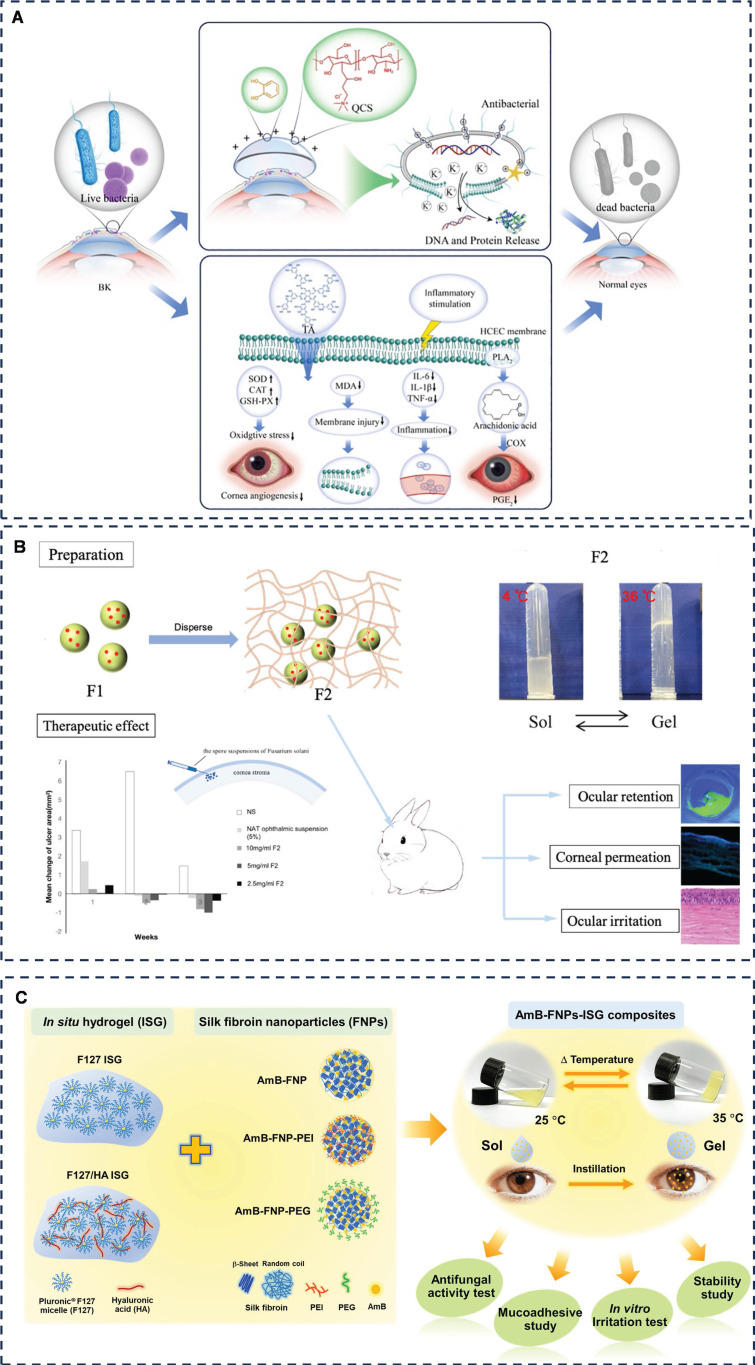
**Representative diagram of adhesive hydrogels applied for eye surface infection. A)** Drug-free contact lens based on quaternized chitosan and tannic acid for bacterial keratitis therapy and corneal repair. Adapted with permission from 68 Copyright 2022 Elsevier. **B)** Thermosensitive tri-block polymer nanoparticle-hydrogel composites as payloads of natamycin for antifungal therapy against fusarium solani. Adapted with permission from 69, Copyright 2022 Dove Medical Press. **C)** Mucoadhesive hybrid system of silk fibroin nanoparticles and thermosensitive *in situ* hydrogel for amphotericin b delivery: a potential option for fungal keratitis treatment. Adapted with permission from 70, Copyright 2024 MDPI.

Bacterial keratitis, a prevalent ocular infection, was addressed by Jiao *et al*. through the development of a unique polyacrylamide (PAM) semi-interpenetrating network hydrogel contact lens dubbed PAM-QCS-TA (**Figure 4A**) 68. This innovative material is synthesized from quaternate chitosan (QCS) and tannic acid (TA), imparting it with antibacterial and antioxidant qualities. The intricate interpenetration of PAM, QCS, and TA, mediated by hydrogen bonding, electrostatic interactions and π-π stacking, results in a hydrogel that exhibits remarkable resistance to swelling and boasts excellent mechanical properties. Moreover, in a rabbit model of bacterial keratitis, this hydrogel displayed pronounced antibacterial, anti-inflammatory, and cornea-repair-promoting effects.

Fungal keratitis (FK) is a symptomatic, rapidly progressive condition that can severely damage the cornea, including ulceration, perforation, and potentially blindness. The primary therapeutic agents currently employed to treat FK include polyene macrolide antibiotics (5% natamycin [NAT] and 0.25% amphotericin B [AmB]), as well as imidazole and pyrimidine antifungal agents. However, these drugs face significant challenges due to their poor solubility and low bioavailability, often resulting in inadequate disease control and severe consequences. To address these limitations, nano drug delivery systems have emerged as a promising approach to enhance drug solubility and permeability. Sha *et al.* have pioneered the development of NAT-loaded triblock polymer nanoparticles embedded in Pluronic® gel (F127 and PEG-PPG-PEG), creating a thermosensitive hydrogel polymer system with adhesive properties (Gel@PLGA-PEI-PEG@NAT) specifically designed for FK treatment (**Figure 4B**) 69. The system increased cohesion through temperature responsive phase transition, and the enhancement of cohesion in this system, together with mechanical chain effect and hydrogen bonding, played a role in ocular surface adhesion, which ultimately achieved the effect of prolonging drug residence time and increasing drug permeability. Notably, in a rabbit model of FK, a 2.5 mg/mL hydrogel system achieved therapeutic efficacy comparable to a 50 mg/mL NAT ophthalmic suspension, significantly enhancing drug bioavailability and reducing the need for frequent dosing. Furthermore, Chomchalao *et al.* have introduced a combination system utilizing filipin protein nanoparticles encapsulating AmB and incorporated into a thermosensitive *in situ* hydrogel (AmB-FNPs ISG) (**Figure 4C**) 70. This system similarly improved cohesion through a temperature-responsive phase transition. Ionic and hydrogen bonds were also formed between the polymer and the mucin of the mucosa, which greatly improved the adhesion of the system. The polymer system was formed at a gel-forming temperature of 35 ± 1 °C, and ocular surface adhesion was maintained for more than 6 hours, which not only prolonged the ocular retention time of AmB, but also reduced the frequency of administration during treatment.

Amit* et al.* designed a cornea-specific cell-penetrating the peptide for the treatment of FK, but the duration of action was only 4 hours. In order to improve the bioavailability and delivery of this peptide, they introduced the cell-penetrating peptide into a gelatin hydrogel system and succeeded in extending the antifungal activity of the biopeptide to 24 hours, which reduces the number of applications and is more favorable for the treatment of FK 71. This study did not elaborate on the adhesion mechanism, but analyzed actin expression in corneal epithelial cells inoculated with soft and hard hydrogels, respectively. The results showed that corneal epithelial cells adhering to the soft hydrogel exhibited prominent stress fibers compared to the hard hydrogel. Here, we hypothesized that it may be mechanical effects such as the pores and flexible surface of gelatin that promote cell adhesion, thus enabling the polymer system to prolong drug action. Adhesive hydrogels have diverse applications, ranging from drug delivery for infectious ocular diseases to assisting in the diagnosis of various ocular surface infections. Swift *et al.* have pioneered two methods for specifically and swiftly detecting and differentiating between Gram-positive, Gram-negative bacteria, and fungi in infected corneas 72. The first method involves a glycerol mono-methacrylate (GAMA) hydrogel swab coupled with a highly branched polymer additive functionalized with vancomycin (VAN), polymyxin (PMX), and amphotericin (AMP) ligands, exhibiting high selectivity for bacterial and fungal strains. The second method utilizes modified commercial contact lenses (carboxylate contact lenses) functionalized with similar ligands, though it is less specific than the first approach. Both methods are based on functionalisation of GAMA swabs or contact lenses with highly branched polymer additives that increase affinity for bacterial or fungal isolates by forming covalent bonds such as amide bonds. These methods rapidly determine the type of infection (Gram-positive, Gram-negative, or fungal) in less than 30 minutes, greatly improving clinical diagnosis and treatment.

Ocular surface infections, though common, are complex eye diseases that require prompt and targeted diagnosis and treatment. However, misuse of antibiotics often leads to atypical presentations, potentially misleading doctors and delaying treatment. While numerous studies have focused on improving drug delivery efficiency, fewer have addressed diagnostic efficiency. Therefore, enhancing diagnostic methods for ocular surface infections represents a promising direction for future research.

#### 3.1.3 Corneal wound surgery

Corneal disease stands as a leading cause of visual impairment and blindness globally. Although corneal transplantation is the most effective treatment for advanced cases, only 5% of patients have access to this procedure due to the scarcity of donors and the associated high costs 73. Furthermore, allogeneic grafts pose inherent risks of immune rejection and infection 74, 75. Beyond corneal transplantation, any intraocular surgery involving corneal incision, such as cataract IOL replacement or vitrectomy, typically requires sutures for closure. However, these sutures inflict additional trauma on corneal tissues, potentially leading to localized inflammation or infection. Moreover, corneal sutures often result in uneven healing, causing astigmatism that can significantly impact visual formation 76.

Grinstaff *et al.* proposed a set of ideal properties for corneal adhesives to address these challenges 76. These include: strong wet adhesion capable of withstanding intraocular pressure (IOP) exceeding 80 mmHg; controlled rheological properties with a viscosity below 100 cP; rapid curing within 30 seconds; swift IOP recovery within 24 hours; refractive index matching the natural cornea (1.42); solute diffusion properties conducive to normal corneal healing (exceeding 2×10^-7^ cm^2^/s for small molecules/nutrients); biocompatibility; elasticity exceeding corneal tissue to prevent astigmatism during healing; provision of a microbial barrier for 2-3 days; and timed removal from the wound, aligning with tissue regeneration through bio-absorption or exudation (ranging from days to months depending on the application).

Biomaterials used as tissue adhesives for corneal sealing and repair can be broadly categorized into two types based on their source: (i) naturally derived materials, such as extracellular matrix components (gelatin, collagen, fibrin) and polysaccharides (hyaluronic acid, chitosan), and (ii) synthetic materials, such as cyanoacrylate and polyethylene glycol (PEG). Each type has its own advantages and disadvantages. Naturally derived polymers generally offer better biocompatibility, but they often exhibit lower mechanical stability and adhesive strength. In contrast, synthetic polymers allow for tailored formulations to meet specific requirements but tend to have poorer tissue regeneration and cell adhesion properties.

**Figure 5:**
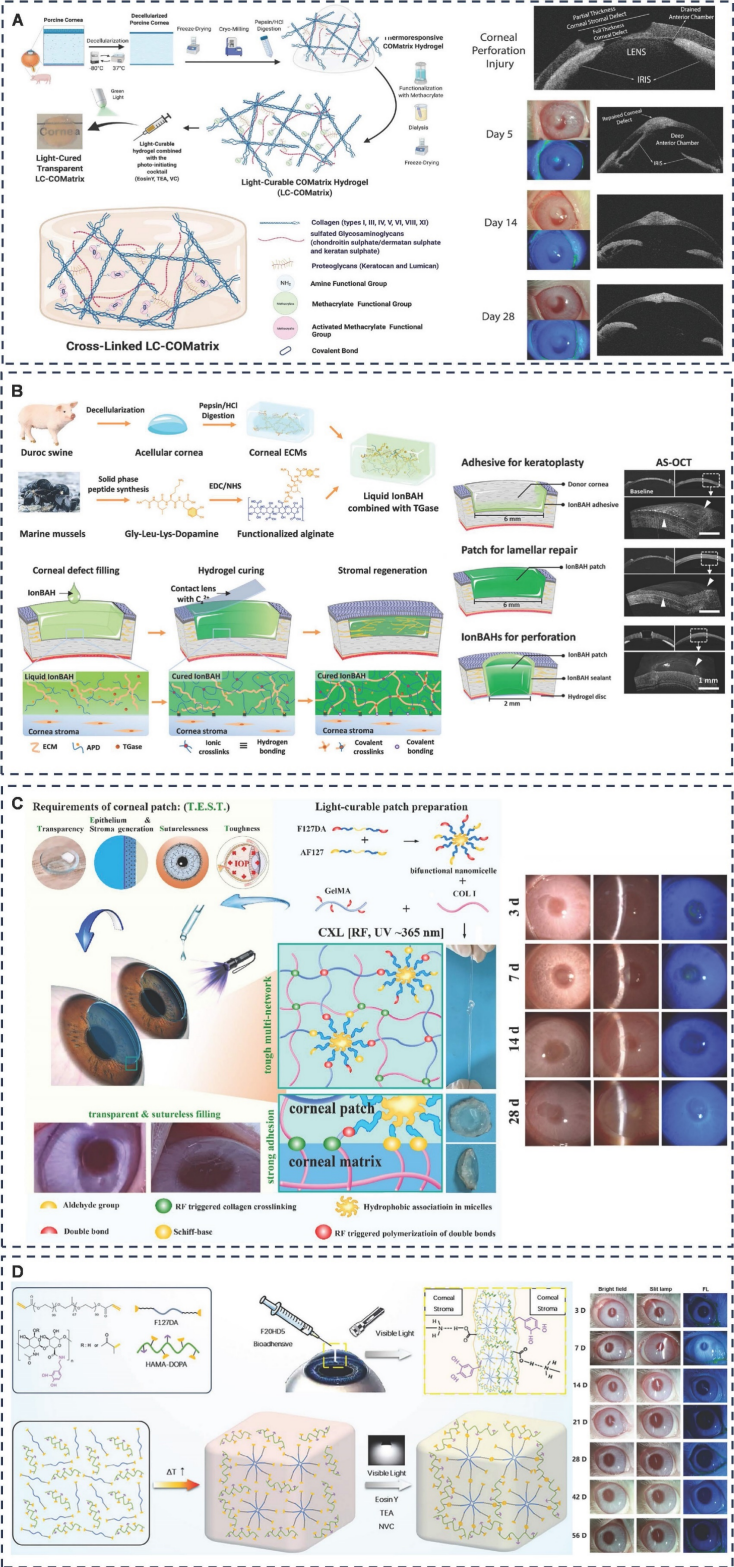
**Representative diagram of adhesive hydrogels applied for corneal wound surgery. A)** A Light-Curable and Tunable Extracellular Matrix Hydrogel for *In Situ* Suture-Free Corneal Repair. Adapted with permission from 78, Copyright 2022 Wiley. **B)** Natural Dual-Crosslinking Bioadhesive Hydrogel for Corneal Regeneration in Large-Size Defects. Adapted with permission from 79, Copyright 2022 Wiley**. C)** A "T.E.S.T." Hydrogel Bioadhesive Assisted by Corneal Cross-linking for *In Situ* Sutureless Corneal Repair. Adapted with permission from 95, Copyright 2023 Wiley. **D)** Photocurable and Temperature-Sensitive Bioadhesive Hydrogels for Sutureless Sealing of Full-Thickness Corneal Wounds. Adapted with permission from 100, Copyright 2024 Wiley.

The extracellular matrix (ECM) of the corneal stroma is a complex network comprising primarily water, collagen, and glycosaminoglycans (GAGs) 77. Researchers have explored various materials and strategies to mimic this ECM for corneal tissue engineering. Yazdanpanah *et al.* developing a light-cured corneal stroma (LC-COMatrix) based on decellularized porcine corneal ECM (DPC-ECM). This material exhibits suitable swelling behavior, biodegradability, and viscosity, and can be crosslinked *in situ* under visible light to significantly enhance biomechanical strength, stability, and adhesion (**Figure 5A**) 78. Crosslinked LC-COMatrix showed strong adhesion to isolated human corneas and effectively closed full-layer corneal perforations and tissue defects. The naturally derived and biocompatible nature of these materials facilitates clinical translation. Zhao *et al.* pioneered the development of an ion-activated bio-adhesive hydrogel (IonBAH) specifically for corneal repair (**Figure 5B**) 79. This innovative hydrogel comprises DPC-ECM, peptide-modified alginate, and transglutaminases (TGases) utilizing the synergistic effect of covalent and ionic bonding of DOPA and TGase-mediated cross-linking to achieve robust adhesion to the recipient cornea. After six months of observation, IonBAH exhibited remarkable results, facilitating rapid regeneration of corneal epithelium, stroma, and nerves, restoring transparency and thickness, thus achieving therapeutic effects comparable to donor corneal transplantation.

Another interesting approach was demonstrated by Fernandes-Cunha *et al.*, who utilized supramolecular noncovalent host-guest interactions between HA-cyclodextrin and HA-adamantane to create shear-thinning HA hydrogels. These hydrogels promoted adhesion and spreading of encapsulated human corneal epithelial cells in *ex vivo* models and improved corneal wound healing *in vivo* 80. In summary, researchers have explored a variety of strategies to mimic the corneal ECM using synthetic and naturally derived materials. These advances hold promise for the development of novel corneal substitutes and adhesives that can effectively repair corneal defects and improve patient outcomes.

Achieving a watertight closure of clear corneal incisions during cataract surgery is crucial for minimizing the risk of infection and other leakage-related complications, as it prevents fluid from infiltrating the eye and thus reduces the likelihood of intraocular infection 81. As early as 1996, a team began investigating the use of fibrin glue to seal corneal incisions after cataract surgery 82. However, due to the toxicity of cyanoacrylates and the heat generated during polymerization, they were not widely used clinically, and further researchers looked to the much less toxic fibrin glue 83, 84. Banitt *et al.* compared the healing effects of fibrin glue (Tisseel®), cyanoacrylate glue (Histoacryl), and suture closure on post-cataract corneal incisions of different widths 85. The researchers found that all three did a good job of sealing corneal incisions created by cataract surgery. But there were differences in maintaining a certain amount of intraocular pressure after surgery. Tisseel® maintained better IOP than sutures when sealing 3-mm incisions, but did not achieve ideal IOP when sealing 4.5-mm or 6-mm incisions. This suggests that it may be caused by the fact that Tisseel® is of human origin and degrades easily.

In recent years, the FDA-approved polyethylene glycol hydrogel Resure® has been proven effective in corneal wound closure in cataract surgery. Studies conducted by Tong *et al.* and Masket *et al.* revealed that Resure® performs comparably to suture closure in sealing cataract surgical wounds, without causing adverse effects such as a foreign body sensation or an increase in surgically-induced astigmatism 20, 86.

Nallasamy *et al.* further investigated the efficacy of Resure® in both routine and complex cataract surgeries, finding that a hydrogel ocular sealant was more suitable due to its ability to provide a more stable positioning for complex multi-intersection adjustable IOLs 87. Spierer *et al.* examined the effectiveness of Resure® in sealing corneal incisions during Descemet's stripping endothelial keratoplasty (DSEK) surgery, both alone and combined with cataract surgery. They concluded that Resure® effectively and rapidly closed corneal incisions in DSEK surgery, with or without cataract surgery, significantly reducing foreign body sensation and inflammation associated with sutures, while also saving surgical time 88. In summary, Resure® sealants with polyethylene glycol (PEG) as the main ingredient, this polymer has excellent biocompatibility and water solubility, and its strong cohesion is one of the reasons for its adhesion. When used on tissue surfaces, they can generate hydrogen bonds and mechanical interlocking effects, resulting in better adhesion. Building on its strong mechanical and adhesive properties, Zhou *et al.* developed an injectable hydrogel based on PEG-lysozyme for repairing corneal stromal defects 89. The NHS group of 4-arm-PEG-NHS reacts with amine groups on corneal tissue, forming amide bonds that serve as the primary adhesive mechanism between the hydrogel and the corneal surface. Additionally, the 4-arm-PEG-NHS and lysozyme mixture maintains suitable fluidity before curing, allowing it to fill irregular defects. This promotes mechanical interlocking adhesion and enables the formation of a highly shape-adaptive hydrogel implant.

Gelatin (Gel), a natural protein polymer approved by the U.S. Food and Drug Administration (FDA), is derived from animal sources through the hydrolytic degradation of type I collagen 90, 91. It boasts high biodegradability, biocompatibility, and non-toxicity. GelMA, a modified version of Gelatin, incorporates meth-acrylamide groups that enable its photoinitiated, free radical polymerization to create covalently crosslinked hydrogels 92. This controlled photoinitiated polymerization renders GelMA a favorable choice for ophthalmic clinical applications, leading to the development of numerous GelMA-based polymer systems.

Sani *et al.* developed GelCORE, a gelatin-based adhesive hydrogel for the swift and enduring repair of corneal interstitial defects, by crosslinking with Type II initiators, Eosin Y, triethanolamine (TEA), and N-vinylcaprolactam (VC) through a free radical polymerization process 93. After just four minutes of photo-crosslinking, this hydrogel forms a robust, transparent, and adherent gel on corneal tissue. Furthermore, its burst pressure test results on isolated rabbit eyes surpassed those of the commercially available Resure® hydrogel. However, the team noted a limitation of GelCORE: its high liquid content, which led to easy diffusion and loss after application, rendering it unsuitable for bonding full-thickness corneal injuries. To address this challenge, the team developed a light-polymerized hydrogel patch based on GelMA (gelatin methacrylate), incorporating hyaluronic acid glycidyl methacrylate (HAGM) and PEG diacrylate (PEGDA) 94. HAGM increased the viscosity of the polymer system, while PEGDA enhanced its flexibility and extensibility, resulting in improved adhesion. The average burst pressure of this new hydrogel was 3-4 times greater than that of Resure® hydrogel. This multi-source polymer system effectively balanced the properties of its components, leading to a more versatile adhesive hydrogel for corneal injury repair.

In addressing the concern for corneal repair following cross-linking surgery in patients with conical corneas, Zhou *et al*. emphasized the need for corneal substitutes to fulfill four critical properties, namely "transparency," "epithelial and stromal production," "suture-free," and "toughness," collectively referred to as "T.E.S.T." Type I collagen plays a pivotal role in the properties of corneal substitutes (**Figure 5C**) 95. To this end, the research team combined collagen type I (COL-I), Pluronic F127 diacrylate (F127DA), aldolized Pluronic F127 (AF127), and gelatin methacrylate (GelMA) to create an advanced bio-adhesive hydrogel substitute rooted in GelMA that fulfills the "T.E.S.T." criteria. The hydrogel's superior tissue adhesion ability stems from the aldehyde groups in the micelles, the corneal cross-linking-induced COL-I connection to the corneal ECM, and the in-situ network formation through light curing. To validate its efficacy, the corneal tissue adhesion of these hydrogels was rigorously evaluated both *in vitro* using porcine corneas and *in vivo* in rabbit models, encompassing lamellar corneal transplantation and the repair and replacement of deep corneal defects. This comprehensive study, leveraging materials approved by the U.S. Food and Drug Administration, holds promising prospects for clinical translation.

Zhao *et al.* also integrated oxidized dextran (ODex) into the rigid photopolymerized GelMA hydrogel, facilitating the formation of a dual network under visible light to bolster its adhesive properties. Further assessment of the adhesion effect, biocompatibility, and potential clinical applications was conducted in a New Zealand rabbit corneal lamellar graft model 96. Wang *et al.* crafted GelMA/HA-NB hydrogels by fine-tuning the ratio of GelMA to butyramide (NB)-modified hyaluronic acid (HA-NB), mirroring the human corneal collagen-to-glycosaminoglycan ratio 97. This hydrogel boasts robust wet tissue adhesion, swift gelation, minimal swelling, and exceptional biocompatibility. A long-term study using the New Zealand rabbit large-diameter corneal defect regeneration model explored the biocompatibility and clinical potential of this ECM-mimicking bonding hydrogel. Qian *et al.* developed a dopamine methacrylamide (DMA)-based adhesive hydrogel, building upon the transparent Gel/DMA base, through oxidative radical polymerization. This hydrogel excels in transparency, adhesion, oxidation resistance, and biocompatibility 98. Notably, the adhesion of Gel/DMA, primarily attributed to the catecholamine groups of DMA, surpasses even clinically used PEG-based adhesives (Evicel). Furthermore, a rabbit corneal stromal defect model demonstrated that this bioadhesive significantly accelerated epithelialization in damaged corneas *in vivo*. Additionally, an adhesive hydrogel (dCor/Gel-PEG) has been developed based on GelMA, combined with decellularized bovine corneal stroma and PEG, to treat corneal defects, offering a new design approach for corneal adhesive hydrogels 99. Shear testing indicates that dCor/Gel-PEG hydrogel achieves a shear strength of approximately 945 ± 23 kPa on dry substrates and 732 ± 29 kPa on wet substrates, surpassing or equaling the adhesive strength of commercial surgical sealants (Evicel at 207.7 ± 67.3 kPa, CoSEAL at 69.7 ± 20.6 kPa, and cyanoacrylate tissue adhesives at 115 ± 22 kPa). This impressive adhesive strength is primarily attributed to the formation of a double network structure that enhances the topology, and covalent bonding between the -CH_2_ groups of PEGDA and the -NH_2_ groups of lysine in dCor.

On a different note, Wang *et al.* successfully synthesized an injectable, light-curable bioadhesive hydrogel (F20HD5) utilizing polyether F127 diacrylate (F127DA) and dopamine-modified hyaluronic acid methacrylate (**Figure 5D**) 100. This hydrogel is specifically designed for the seamless closure of total corneal incision wounds. The study revealed that F20HD5 exhibits remarkable transparency, optimal viscosity, biodegradability, and exceptional biocompatibility. It effectively seals various types of rabbit corneal wounds, maintaining corneal curvature and clarity even after 56 days of follow-up.

The cornea is a resilient tissue with tightly arranged structures, so materials suitable for corneal adhesion must also exhibit good mechanical compliance with the underlying tissue. Zhang *et al.* adjusted the crosslinking degree of alginate by MA (AlgMA) crosslinking and calcium ion crosslinking, then combined it with ODex and dendritic polymers to form a triple-crosslinked double-network hydrogel for corneal tissue bonding 101. Shear tests on regenerated cellulose membranes and biological amniotic membranes revealed that calcium ion chelation improved adhesion by twofold, and the triple-crosslinked double-network hydrogel further enhanced this by an additional factor of one. This study suggests that increasing the crosslinking density of polymers and designing multiple crosslinking, interpenetrating network polymers are effective strategies to improve corneal adhesives. However, a limitation is that multiple crosslinking often affects the transparency of the polymer. Transparency is critical for corneal adhesives, so balancing crosslinking density and adhesive strength, while considering tissue characteristics, is an important area for further research.

Current research predominantly centers on enhancing adhesion, biocompatibility, and promoting corneal healing. However, interstitial corneal fibrosis, a prevalent cause of corneal injury, inflammation, and vision loss post-surgery 102, remains a challenge. While researchers have formulated polymer hydrogels to prevent corneal scarring and stromal fibrosis in lamellar corneal transplants 103, these hydrogels often lack robust adhesive properties. Therefore, future research on corneal adhesives should aim to not only ensure strong adhesion but also inhibit corneal stromal fibrosis and scar formation, ultimately leading to improved postoperative vision outcomes.

#### 3.1.4 Refractive surgery wounds

Excimer laser *in situ* keratomileusis (LASIK) is a widely practiced surgical procedure for correcting refractive errors 3. Since 1995, over 8.5 million individuals in the United States have undergone refractive surgery, with a total of 13 million eyes treated via excimer laser keratomileusis 104,64. However, corneal wound healing following LASIK surgery is a slow and incomplete process, often taking 3 to 4 years to complete. Microscopic examinations reveal that the LASIK flap heals through the development of oligocellular primary mesenchymal scarring at the central and paracentral interfaces, and multicellular fibrous mesenchymal scarring along the flap's edges. In some cases, inward growth of the corneal epithelium has been reported following LASIK, typically when surgical intervention is inadequate. Conversely, when the procedure is coupled with the use of ocular sealing hydrogels, such as ReSure®, a significant reduction in this inward growth has been observed 105-107.

Despite the limited research in this field, the escalating number of patients seeking refractive error correction through excimer laser corneal surgery underscores the need for ocular adhesives. Swift and precise adhesive bonding is more effective than natural corneal adhesion in enhancing corneal alignment restoration and minimizing postoperative complications, including astigmatism and dry eye. Given that laser surgery can damage subepithelial corneal nerve fibers, the development of adhesive hydrogels that promote corneal nerve growth remains a promising area of investigation.

#### 3.1.5 Conjunctival defect

The conjunctiva, a vital structure of the ocular surface, plays a crucial role in maintaining ocular health. Its cup cells secrete mucin, a primary component of the tear film's mucin layer, essential for lubricating and protecting the eye. Additionally, the conjunctiva functions as a vital immune tissue, safeguarding the eye from external threats while housing numerous immune cells, including macrophages and CD4/CD8-positive T cells. However, in cases of significant conjunctival defects resulting from ocular chemical burns, Steven-Johnson syndrome, pterygium, or conjunctival swelling, conjunctival scarring and contracture often arise due to the tissue's loose structure and abundant fibroblasts. Here, hydrogels emerge as promising therapeutic aids, mimicking the extracellular matrix (ECM) to facilitate conjunctival epithelial repair and providing scaffolds to guide the healing process.

**Figure 6:**
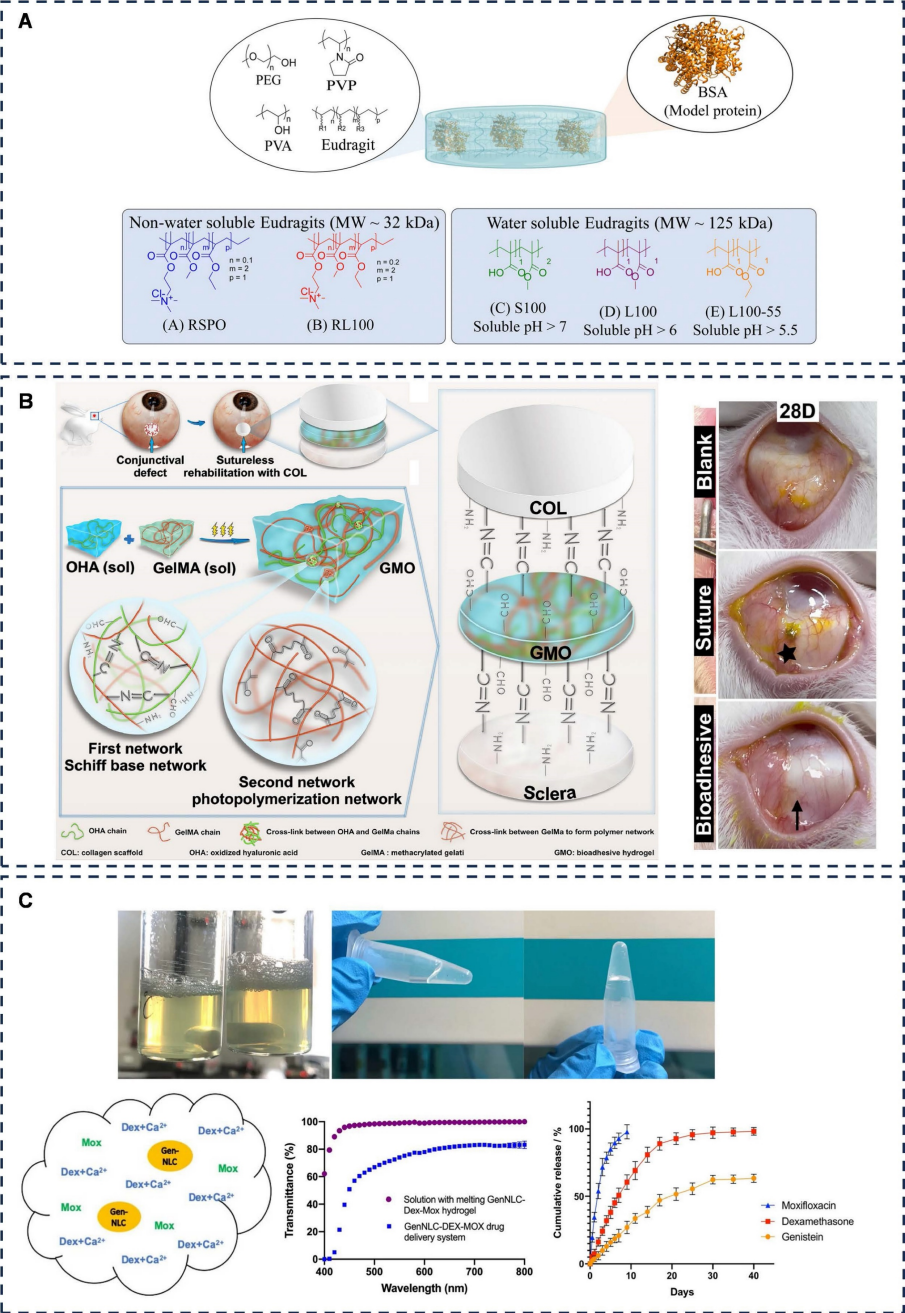
**Representative diagram of adhesive hydrogels applied for conjunctival defect and cataract. A)** The role of Eudragit® as a component of hydrogel formulations for medical devices. Adapted with permission from 110, Copyright 2023 Royal Society of Chemistry. **B)** Sutureless transplantation using a semi-interpenetrating polymer network bioadhesive for ocular surface reconstruction. Adapted with permission from 114, Copyright 2022 Elsevier. **C)** Thermoresponsive genistein NLC-dexamethasone-moxifloxacin multi-drug delivery system in lens capsule bag to prevent complications after cataract surgery. Adapted with permission from 128, Copyright 2021 Nature Portfolio.

Eudragits® polymers, renowned for their gastrointestinal mucosal adhesion 108 and ocular drug delivery capabilities 109, have shown potential in conjunctival applications. Esporrín-Ubieto *et al.* have investigated the adhesion of Eudragits®-based hydrogels to the conjunctiva, demonstrating the feasibility of creating hydrogels with enhanced conjunctival adhesion for drug delivery and tissue repair purposes by optimizing the ingredient ratios (**Figure 6A**) 110.

Autologous conjunctival grafts are presently the gold standard for preventing pterygium recurrence, often affixed to the sclera via sutures. However, recent advancements have introduced fibrin glue as a suture-free alternative, offering reduced inflammation, lessened pain, expedited surgery time, and potentially lower recurrence rates 111, 112. Bondalapati *et al.* experimented with ReSure®, a corneal sealant commonly used in cataract surgery, during amniotic membrane grafting in pterygium surgeries. Notably, after excision in nine eyes, they observed no graft dislocation or failure, and there were no recurrences during follow-up 113. This promising finding suggests that ReSure® could serve as a potential amniotic adhesion sealant in suture-free pterygium surgeries.

Furthermore, Liu *et al.* have devised a semi-interpenetrating polymer network (sIPN) tissue-adhesive hydrogel, GMO, comprising GelMA and an interfacial initiator (OHA), for ocular surface reconstruction (**Figure 6B**) 114. Their study on conjunctival defects in New Zealand rabbits demonstrated that GMO, paired with collagen scaffolds, promoted conjunctival epithelial hyperplasia and repair, without postoperative scarring or conjunctival contracture, outperforming suturing methods. This sIPN-based bioadhesive, characterized by the molecular-level penetration of macromolecules into polymer networks, offers both robust mechanical strength and adjustable composition ratios for tailored adhesion 115. Compared to interpenetrating polymer networks (IPNs), sIPNs provide an enhanced combination of strength and flexibility, making them a promising material for ophthalmic applications. Zheng *et al.* developed an elastic and resilient hydrogel patch named APTF, designed with strong cohesion to ensure robust adhesion 116. APTF is primarily structured with poly (ethylene glycol) diacrylate (PEGDA) as its main backbone. Its elasticity and toughness are achieved through ionic interactions between N-hydroxysuccinimide (NHS)-conjugated alginate (Alg-NHS) and Fe3+, as well as reversible hydrogen bonding between PEGDA and tannic acid (TA). The adhesive strength of APTF is further enhanced by the wet adhesion properties of TA (via hydrogen and covalent bonds) and the covalent bonding between NHS groups and amine groups on tissue surfaces. In lap shear tests, APTF demonstrated robust adhesion (77.28 ± 3.39 kPa), securely attaching to corneal (19.61 ± 3.26 kPa) and conjunctival tissues (11.87 ± 2.57 kPa). Moreover, APTF can be easily removed from these tissues using a urea solution, offering a promising approach for ocular surface and conjunctival repair that accounts for removal considerations. This design shows significant potential for clinical application.

The conjunctiva, though not directly involved in the formation of the optical pathway, plays a crucial role in ocular surface immunity. However, significant conjunctival defects often result in scarring due to the tissue's laxity, ultimately leading to eyelid contracture that impacts vision and aesthetics. Consequently, the development of soft and flexible hydrogel polymers tailored for conjunctival tissue is imperative. These polymers must support conjunctival epithelial repair while inhibiting scar formation. Furthermore, given the direct contact between the eye and the external environment, as well as the potential for microbial colonization on the ocular surface, hydrogels with improved anti-inflammatory and antimicrobial properties are essential. Currently, hydrogels used for conjunctival defect repair primarily focus on adhesion and biocompatibility. In the future, multifunctional adhesive hydrogels loaded with antifibrotic, anti-inflammatory, and antimicrobial bioactive molecules or drugs could be designed to effectively inhibit conjunctival scar formation and enhance anti-inflammatory and antimicrobial effects.

In summary, the focus of research on adhesive hydrogels for ocular surface diseases has shifted from pure adhesion, which binds tissues together, to the design of biocompatible adhesive hydrogels with a variety of functions, including, but not limited to, facilitating repair, loading of drugs, inhibition of oxidative stress, and inhibition of inflammatory responses. Next, we will present research on adhesive hydrogels for the treatment of the corresponding diseases in four areas of ocular diseases, including cataracts, glaucoma, vitreoretinopathy, and open ocular trauma (**Table 2**).

### 3.2 Cataracts

Cataract stands as the foremost cause of blindness globally. Presently, the primary treatment modality for cataracts is surgical, encompassing extracapsular extraction, lens ultrasonic emulsification techniques, and intraocular lens (IOL) replacement. These procedures are highly effective in significantly enhancing vision. Nonetheless, as cataract surgery involves intraocular manipulation, it can occasionally lead to severe complications such as intraocular inflammation, infection, or posterior capsule opacification (PCO) 5. Given that cataract surgery necessitates a corneal incision, and we have previously delved into hydrogels for corneal adhesion, this section will primarily delve into the research surrounding adhesive hydrogels for drug delivery post-cataract surgery.

After cataract surgery, antibiotic and glucocorticoid drops are routinely administered to safeguard against intraocular infections by providing antibacterial and anti-inflammatory treatments. In order to enhance patient compliance during the postoperative period, numerous efforts have been made to devise ocular drug delivery systems as alternatives to traditional eye drops. These include the anterior chamber implants such as Surodex® 117-120, DEXYCU® 121 and IBI-10090® 122. The positive outcomes of clinical trials have bolstered confidence in the field of ocular anti-inflammatory drug delivery, leading researchers to explore the delivery of diverse drug types.

Polyether F127 is a synthetic A-B-A triblock copolymer with the molecular structure of poly(ethylene glycol)-poly(propylene glycol)-poly(ethylene glycol) 123. The poly (propylene glycol) chain segments in the middle of the molecule are relatively hydrophobic while the poly (ethylene glycol) chain segments at the ends are relatively hydrophilic. This hydrophilicity and hydrophobicity make it self-assembling into nanomembranes in water, which is often used for solubilisation and loading of hydrophobic drugs. In addition, F127 has reversible temperature-raising thermogenic gelation properties 27, 124. Sapino *et al.* developed an ophthalmic nanocomposite hydrogel drug delivery system consisting of solid lipid nanoparticles loaded with dodecyl cefuroxime (dCEF) and an oil-in-water microemulsion with Pluronic® F127 (20% w/v) 125. This system, with increased cohesion due to hydrogen bonding at elevated temperatures, formed an in-situ hydrogel with some viscosity at the ocular surface to achieve a slow-release effect, thus prolonging the efficacy of CEF and enabling its sustained release for up to one week.

Posterior cataract is a common complication after cataract IOL implantation 126. Liu *et al.* introduced a nanostructured lipid carrier (NLC) that prevents intracapsular collagen deposition and lens fibrosis 127. Yan *et al.* further advanced this area of research by combining a nanostructured lipid carrier loaded with genistein flavonoid (GenNLC) with a F127/F68 hydrogel containing dexamethasone (Dex) and moxifloxacin (Mox) (**Figure 6C**) 128. The result produced a temperature-sensitive *in situ* hydrogel suitable for injection into the anterior chamber after cataract surgery. The system reduces inflammation, prevents infection and minimises posterior capsule opacity. The system forms a moderately viscous gel at approximately 32°C, providing the operator with sufficient time to perform anterior chamber injections, while ensuring that the gel forms rapidly upon entry into the lens capsule and is less prone to dislodgement, helping to reduce the incidence of posterior cataract and endophthalmitis.

For post-cataract surgery anti-infective treatment, patient compliance holds paramount importance, and the utilization of adherent hydrogels has the potential to minimize the need for postoperative medications and enhance patient adherence. Nevertheless, the precision of lens selection and measurement before the procedure is also essential for successful visual recovery 129, 130. Consequently, the application of adhesive hydrogels in cataract surgery should not be confined to intra- and post-operative phases alone. Looking ahead, researchers can explore integrating these hydrogels with biosensors to develop ocular biosensors capable of measuring axial and corneal curvature, facilitating lens calculations. This approach could pave the way for personalized and precise designs that contribute significantly to enhancing visual function.

### 3.3 Glaucoma

Glaucoma, a complex, progressive neurodegenerative disorder, is marked by the deterioration of the optic nerve and the loss of retinal ganglion cells, ultimately resulting in vision impairment and the narrowing of the visual field. Statistically, glaucoma stands as one of the four primary culprits behind blindness globally, yet it is a condition that can be managed and intervened with medication and surgical procedures 131. Among the prevalent treatment options for glaucoma patients, the administration of medications to regulate intraocular pressure (IOP) within healthy limits is commonplace. This approach necessitates the regular application of eyedrops to lower IOP and hinder disease progression. However, the frequent use of eyedrops can be cumbersome for patients, and the preservatives present in these drops have the potential to damage the ocular surface. To mitigate this issue, numerous studies have been undertaken, aiming to devise methods for sustained release and dose reduction through the formation of *in situ* hydrogels with adhesive properties on the ocular surface.

Dendritic polymers were first invented and successfully synthesized by Dr. Tomalia DA, an American chemist, in the early 1980s 132. Nowadays, dendritic polymers are not only used as delivery carriers for bioactives and drugs, but they are also viscous and have been subjected to photocrosslinking and nucleophilic reagent-electrophilic reagent crosslinking by a number of researchers to create adhesive, transparent, elastic, hydrophilic and soft hydrogels 76, 133-137. For the treatment of glaucoma, Mishra *et al.* developed a polypropyleneimine (PPI) dendritic polymer nanostructure loaded with acetazolamide (ACZ) for the treatment of glaucoma 138. The polymer system produced adhesion through hydrogen bonding and peripheral functional groups (amine groups) of the dendritic molecule, which prolonged the ocular retention time of ACZ and enhanced the IOP-lowering effect.

**Figure 7:**
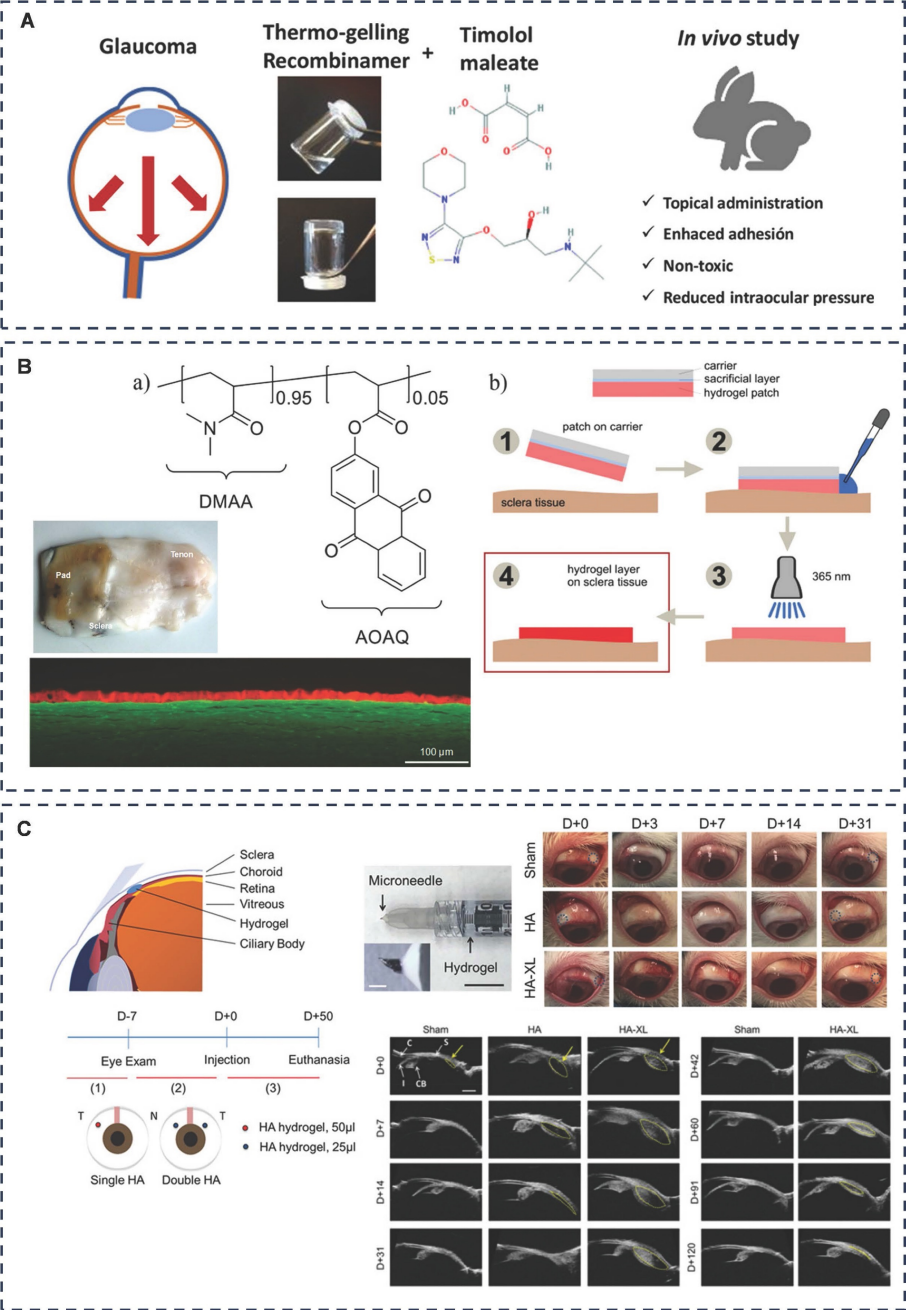
**Representative diagram of adhesive hydrogels applied for glaucoma. A)** Self-assembling elastin-like hydrogels for timolol delivery: development of an ophthalmic formulation against glaucoma. Adapted with permission from 139, Copyright 2017 American Chemical Society. **B)** Prevention of ocular tenon adhesion to sclera by a PDMAA polymer to improve results after glaucoma surgery. Adapted with permission from 142, Copyright 2020 Wiley. **C)** Drug-free, nonsurgical reduction of intraocular pressure for four months after suprachoroidal injection of hyaluronic acid hydrogel. Adapted with permission from 143, Copyright 2021 Wiley.

Fernández-Colino *et al.* evaluated thermosensitive elastin and silk elastin-like recombinants as innovative pharmaceutical dosage forms for topical administration of timolol, a system that can turn into a gel at physiological temperatures and adhere to the ocular surface (**Figure 7A**) 139. The specific mechanism of adhesion was not mentioned in this study, and we hypothesized that it is the balance between the cohesive and adhesive forces of the liquid in the presence of temperature that produces the adhesion effect.

Yang *et al.* designed a novel hybridized polyamidoamine (PAMAM) dendritic polymer hydrogel/poly (lactic acid-glycolic acid co-polyester) (PLGA) nanoparticle platform (HDNP) for the co-administration of two anti-glaucoma drugs, brimonidine and timolol maleate. These two IOP-lowering drugs can be maintained *in vitro* for 28-35 days, and a single application in a rabbit model can maintain the IOP-lowering effect for 4 days 140. In this system, the partially crosslinked PAMAM dendritic polymer G3.0-PEG-acrylate carries a large number of amine groups, which create mucosal adhesion through covalent bonding and enhance interaction with the ocular surface cornea and conjunctiva. This study significantly reduced the dosing frequency of topical formulations and helped improve long-term patient compliance.

Another viable treatment option for glaucoma is surgical intervention, specifically filtering glaucoma surgery, which is often recommended for various glaucoma types. However, a common post-surgical complication is scarring, often triggered by the disruption of the blood-atrial fluid barrier, and excessive scarring can ultimately result in fistula occlusion 141. To address this challenge, Martin *et al.* have formulated a hydrogel polymer comprising 2-acryloyloxyanthraquinone (AOAQ) and dimethylacrylamide (DMAA) (**Figure 7B**) 142. This polymer serves as an effective barrier against fibroblasts, thereby preventing scar tissue formation at the surgical site. Their team has discovered that this polymer exhibits superior adhesion to isolated porcine sclera and is now poised to test its application on rabbit sclera. This study aims to validate the feasibility of the surgical procedures and demonstrate the long-term adhesion effectiveness of this innovative polymer pad.

Chae *et al.* designed a microneedle injection of an *in situ* molded hydrogel for the treatment of glaucoma by lowering intraocular pressure (IOP) in a drug-free, non-surgical manner (**Figure 7C**) 143. The system (HA-XL) consists of thiol-based modified hyaluronic acid (HA-SH) and polyethylene glycol diacrylate (PEGDA.) HA-XL is viscous, and when injected into the suprachoroidal space (SCS), the hydrogel adheres and stays there to keep the SCS open by solubilizing, thus lowering IOP in patients with glaucoma for up to 4 months. The research team is now working to extend the duration of efficacy by modifying the polymer material (hyaluronic acid) with a view to achieving at least 6 months of efficacy. This would coincide with many patients' visits to the clinic.

The critical issue with glaucoma lies in the prolonged elevation of intraocular pressure (IOP), which can trigger optic nerve atrophy in the fundus. Consequently, adhesive hydrogels play a therapeutic role in glaucoma by effectively reducing IOP. This is achieved through their adhesive properties, improved drug retention for extended drug action, and assistance in establishing ocular aqueous drainage channels. Glaucoma patients necessitate ongoing IOP monitoring, yet these measurements are often conducted in hospitals. Although implantable IOP monitoring sensors exist, they are associated with adverse reactions such as anterior chamber inflammation and implantation trauma 144. To address this, future research could focus on developing a miniature, real-time IOP monitoring biosensor that integrates the adhesive, biocompatible, and multifunctional properties of viscous hydrogels. This innovative device would significantly contribute to the management of patients with chronic glaucomatous eye disease.

### 3.4 Vitreoretinopathy

The retina is an important tissue structure in the eye that converts light signals into neuroelectric signals. With its fine and fragile tissue structure and high oxygen demand, the retina is particularly susceptible to oxidative damage, and oxidative stress damage to retinal tissue, especially that caused by reactive oxygen species (ROS), is present in many diseases 145, 146.

**Figure 8:**
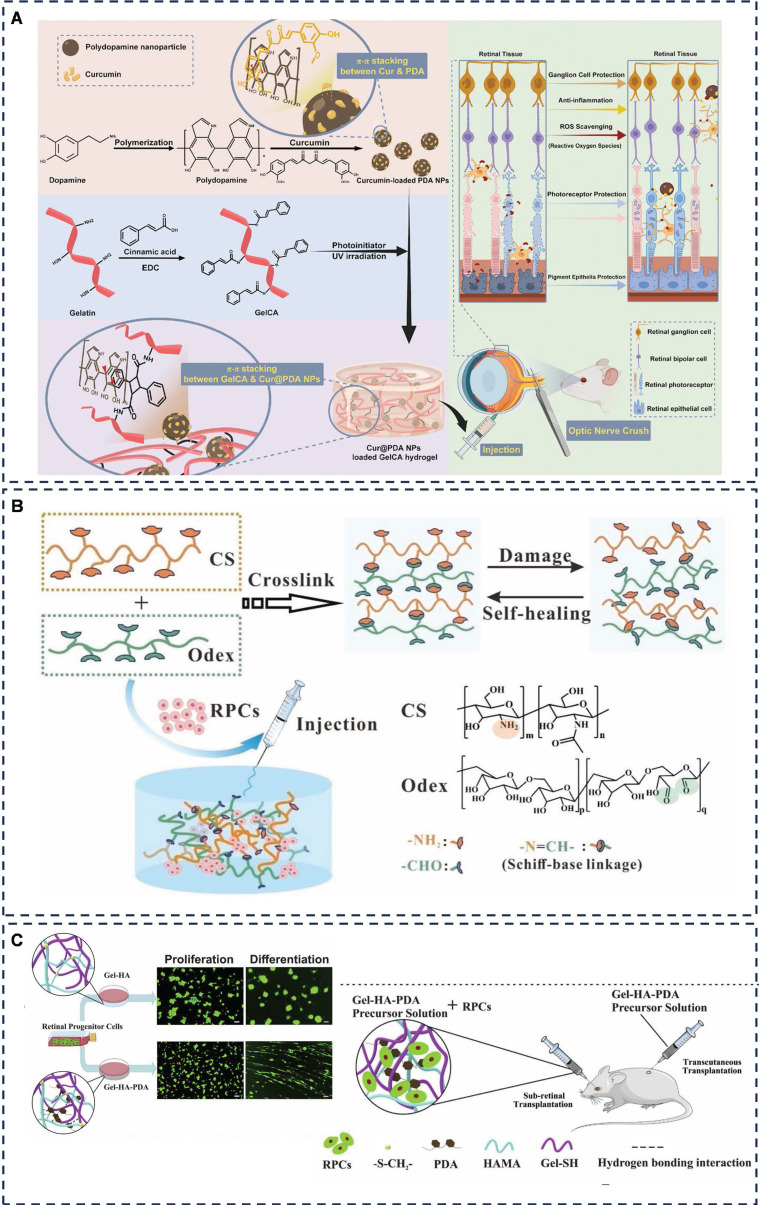
**Representative diagram of adhesive hydrogels applied for retinal regeneration. A)** Injectable, antioxidative, and tissue-adhesive nanocomposite hydrogel as a potential treatment for inner retina injuries. Adapted with permission from 147, Copyright 2024 Wiley. **B)** Enhanced proliferation and differentiation of retinal progenitor cells through a self-healing injectable hydrogel. Adapted with permission from 153, Copyright 2019 Royal Society of Chemistry. **C)** Mussel-inspired injectable hydrogel and its counterpart for actuating proliferation and neuronal differentiation of retinal progenitor cells. Adapted with permission from 157, Copyright 2019 Elsevier.

To combat ROS-related challenges, Liu *et al.* constructed a photocrosslinked, injectable, multifunctional nanocomposite hydrogel, Cur@PDA@GelCA, consisting of cinnamic acid crosslinked gelatin GelCA and curcumin-rich dopamine nanoparticles (**Figure 8A**) 147. This innovative hydrogel showed better biocompatibility, stronger tissue adhesion and oxidative stress inhibition in a mouse retinal injury model. In this system, adhesion was mainly generated by PDA and Cur@PDA NP with amines and thiols on the tissue surface through π-π stacking and hydrogen bonding. This study highlights the promising biomedical applications of adhesive hydrogels in ophthalmology and regenerative medicine, showcasing their versatility and potential impact in these crucial medical fields.

In retinal degenerative diseases such as age-related macular degeneration and retinitis pigmentosa, retinal neurons fail to regenerate. Chen *et al.* tackled this challenge by introducing a novel adhesive-based, protein-free synthetic hydrogel formulated from poly(2-acrylamido-2-methylpropanesulfonic acid sodium salt) (PNaAMPS) and poly(N,N-dimethylacrylamide) (PDMAAm) 148. This hydrogel serves as an RPE cell culture matrix, enabling *in vitro* cultivation of human RPE cell monolayers with low ROS levels, aiming for potential intravitreal placement to foster retinal repair in the future.

While retinal progenitor cell (RPC) transplantation has been proposed to decelerate disease progression 149, 150, its efficacy faces limitations due to the restricted proliferation and differentiation of RPCs post-transplantation. Solid polymer scaffolds, although enhancing RPC proliferation in mice, suffer from issues such as disorganized cell delivery, reduced cell survival, and lack of adaptability and tight integration with the retina 151, 152. Addressing these challenges, Jiang *et al*. developed a self-healing injectable hydrogel, CS-Odex, utilizing chitosan hydrochloride (CS) and oxidized dextran (Odex) as its base (**Figure 8B**) 153. This hydrogel, adhering through dynamic Schiff bases, exhibits high biocompatibility and stimulates AKT and ERK pathways, thereby promoting RPC proliferation and differentiation.

Inspired by the adhesive and proliferative properties of polydopamine (PDA), which promotes synaptic growth, gel-based scaffolds that enhance stem cell proliferation 154, 155, and hyaluronic acid (HA), a key component of the subretina 156. Tang *et al.* devised Gel-HA-PDA, a highly biocompatible and adhesive injectable hydrogel (**Figure 8C**) 157. This hydrogel significantly enhanced the proliferation, migration, and neuronal differentiation (*e.g.*, photoreceptors) of RPCs, presenting a novel biomaterial platform for RPC transplantation-based therapies.

As we age, the vitreous body liquefies, causing it to detach from the retinal surface, resulting in retinal breakage. Liquefied vitreous can enter and accumulate in the subretinal space between the retinal neuroepithelium and retinal pigment epithelium (RPE), leading to fornix retinal detachment (RRD) 158. RRD can be reattached by surgical removal of the vitreous and filling with silicone oil, but the rate of first retinal attachment is about 85% ± 11% 159-161. On the other hand, postoperative proliferative vitreoretinopathy (PVR) may occur due to the presence of a retinal tear 162.

**Figure 9:**
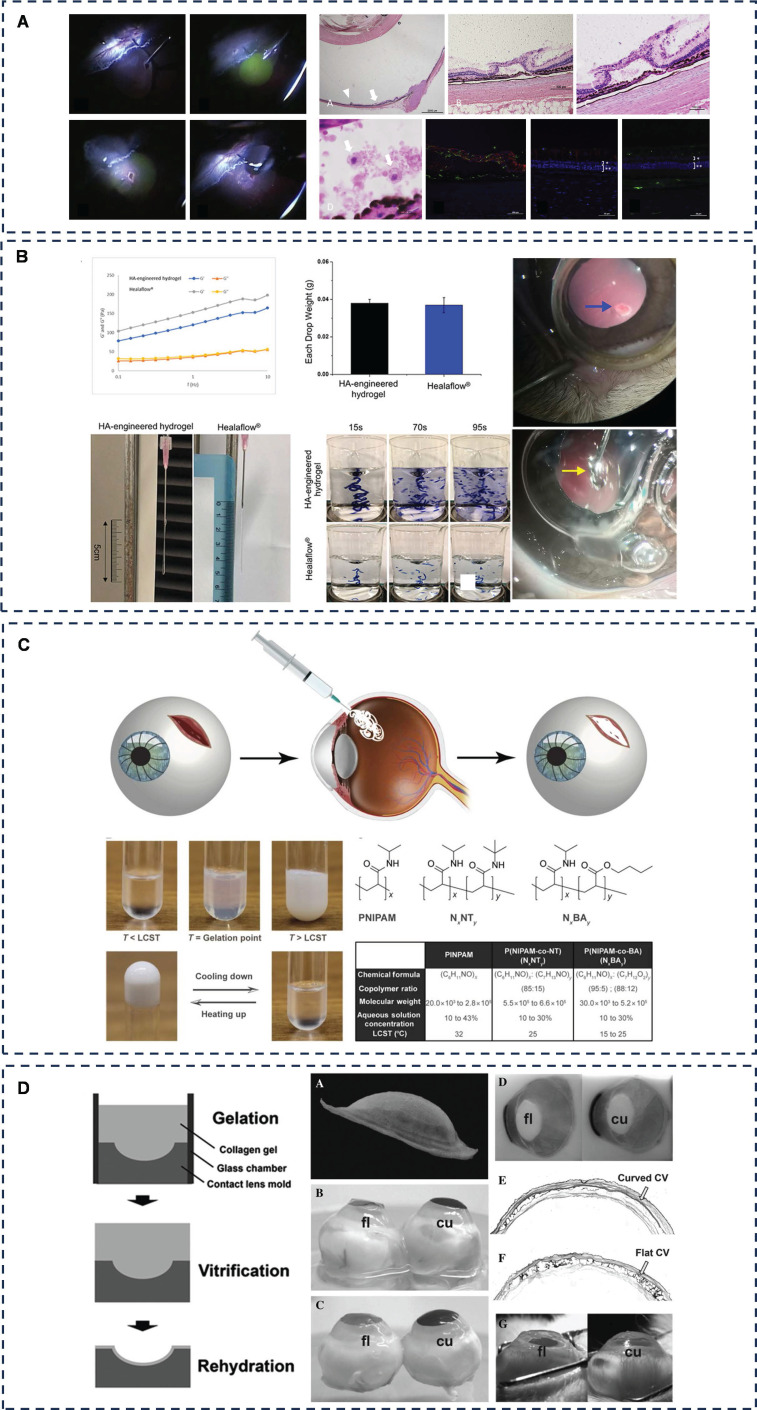
**Representative diagram of adhesive hydrogels applied for retinal detachment and open eye trauma. A)** Patching retinal breaks with polyethylene glycol-based synthetic hydrogel sealant for retinal detachment in rabbits. Adapted with permission from 164, Copyright 2018 Elsevier. **B)** Biocompatibility and efficacy of a linearly cross-linked sodium hyaluronic acid hydrogel as a retinal patch in rhegmatogenous retinal detachment repairment. Adapted with permission from 166, Copyright 2022 Frontiers. **C)** A reversible thermoresponsive sealant for temporary closure of ocular trauma. Adapted with permission from 171, Copyright 2017 American Association for the Advancement of Science. **D)** Application of a collagen-based membrane and chondroitin sulfate-based hydrogel adhesive for the potential repair of severe ocular surface injuries. Adapted with permission from 172 , Copyright 2014 Oxford Academic.

Fibrin glue has been mentioned in corneal wound repair and can exert better tissue adhesion through mechanisms such as mechanical interlocking and ionic bonding. In retinal laceration repair, Tyagi *et al.* conducted a clinical trial of fibrin glue as a repair agent for idiopathic macular laceration, and the results suggest that fibrin glue can be used in patients who have difficulty with postoperative fixation after retinal laceration repair surgery 163. Hoshi *et al.* developed two absorbable PEG hydrogels for the treatment of RD and verified in a rabbit RD model that both PEG hydrogels adhered firmly to the rabbit retina and that FocalSeal treatment restored retinal function after 28 days of follow-up (**Figure 9A**) 164, 165. Zheng *et al.* focused on another FDA-approved hydrogel, hyaluronic acid. They designed an injectable HA-engineered hydrogel based on Healaflow®, a commercially available retinal patch, with physical properties such as pH, osmolality, specific gravity, and refractive index consistent with those of Healaflow®, and strong adhesion through hydrogen bonding, ionic bonding, and mechanical interlocking (**Figure 9B**) 166. *In vitro*, it maintains adhesion to RRD patients for 14 days and adheres to the retina. In a rabbit model of RRD, HA-engineered hydrogel was shown to adhere well to the retina, and at 3-month follow-up, it helped the retina to completely reattach and regain function without the need for expanding gas or silicone oil endothelial tamponade. The results of the current study are a boon to clinical RRD patients, but further long-term follow-up and clinical studies are needed.

Given the crucial role of the retina in converting visual signals into electrical impulses, its treatment demands materials of exceptional biocompatibility. In designing such materials, natural substances like hyaluronic acid, gelatin, sodium alginate, and chitosan are prime candidates due to their high biocompatibility. Adjusting the ratios of these substances can yield polymer systems with dual or multiple networks, balancing flexibility and rigidity. To further enhance retinal nerve regeneration, bioactive proteins like elastin and filipin can be incorporated. These proteins form robust bonds with amino acids on tissue surfaces through hydrogen bonds, π-π interactions, and electrostatic forces, fostering stronger adhesion between tissues and cells 167. Moreover, in evaluating retinal function restoration, while the retinal electrophysiological test remains a cornerstone, advancements can be made by introducing additional tests. Designing full-field photosensitivity threshold tests (FST), multi-luminance mobility tests (MLMT), and other animal-specific evaluations would greatly enrich our means of assessing retinal function.

### 3.5 Open eye trauma

The eye, a closed cavity under high internal pressure, is susceptible to open trauma caused by external forces, sharp objects, or explosions. Although infrequent in urban settings, such trauma can significantly impair vision and predispose to intraocular infections, drastically reducing patients' quality of life. Immediate treatment involves thorough debridement and wound closure using sutures and adhesives. However, currently approved adhesives like fibrin glue 168, 169 and cyanoacrylates 84, 170 fall short due to their cumbersome handling, inadequate adhesive strength, and high toxicity, rendering them unsuitable for open ocular trauma.

Bayat *et al.* introduced an injectable, non-biodegradable liquid sealant formulated from a physically crosslinked copolymer of N-isopropylacrylamide and butyl acrylate (**Figure 9C**) 171. The sealant can be modulated to form a gel at body temperature that adheres to the surface of the eye by mechanical interlocking, diffusion, hydrogen and covalent bonding to effectively seal ocular wounds. Once gelled, it restores intraocular pressure (IOP) and can be removed non-invasively for further treatment within a few days by changing the temperature without damaging the surrounding tissues.

Chae *et al.* developed a viscous hydrogel comprising chondroitin sulfate-polyethylene glycol (CS-PEG) and a collagen vitreous gel (CV) membrane, infused with antibiotics (**Figure 9D**) 172. This CS-PEG-CV biobandage has antimicrobial properties and has some adhesion properties due to hydrogen bonding and mechanical interlocking, and can be used as a biological dressing after ocular trauma. In addition, this dressing is transparent and can close larger corneal wounds without the need for surgical microscopy, making it suitable for emergency situations or battlefields. Future research aims to design bandages of varying sizes and investigate their long-term preservation efficacy.

Bhattacharjee *et al.* addressed both corneal injury closure and infection control by designing an antimicrobial adhesive hydrogel sealant 173. The aldehyde groups in polyglucose aldehyde (PDA) interact with amino groups on tissue proteins, promoting hydrogel adhesion to the tissue surface. Corneal burst pressure tests showed that the BacSeal-3 hydrogel, when used to seal corneal incisions, can withstand pressures comparable to those of cyanoacrylate adhesives (~68 kPa) and demonstrates excellent biocompatibility and antimicrobial effectiveness.

Open ocular trauma, predominantly encountered in war or emergency medical scenarios, demands swift and robust sealing of the eye using adhesive materials with exceptional adhesion properties. This not only involves achieving a dense closure within a short time frame but also necessitates the evaluation of adhesion under diverse environmental conditions, spanning high temperatures, dryness, humidity, underwater settings, and low temperatures. Therefore, enhancing the comprehensive adhesion capability stands at the forefront of designing adhesive hydrogels for this condition. To achieve this, bionic polymeric materials can be crafted, drawing inspiration from mussel-like marine organisms like barnacles and abalone, or by incorporating adhesive agents like DOPA, tannic acid, and tea polyphenols. Alternatively, hydrogel networks can be formulated by modifying reactive groups, such as double bonds, and initiating free radical polymerization on the surface. This approach enables the design of network structures that yield exceptionally strong interfacial adhesion, crucial for effective wound sealing in open ocular trauma.

## 4. Summary and outlook

The ocular surface environment is inherently dynamic and humid, constantly subjected to external factors like wind, tears, dust and varying temperatures. Additionally, mechanical interactions between eyelids and the eyeball during regular activities like blinking, eye rubbing, and sleeping can leave the ocular surface vulnerable to wounds without adequate rest and protection. These complexities pose significant challenges in the design and application of adhesive hydrogels for the ocular surface. To address these challenges, researchers have developed bi- or multi-polymer network hydrogels (interpenetrating or semi-interpenetrating) by incorporating rigid and flexible polymer hydrogels. By adjusting the ratio of these two components, with or without the introduction of adhesive groups like tannic acid and DOPA, they have found formulations that are suitable for ocular surface tissues, further enhancing the overall adhesion of the system 100, 114, 147, 153. Inspired by the asymmetric structure of the peritoneum, Liang *et al.* have successfully designed and prepared PVA hydrogels (JPVA) with a "Janus" porous structure using a top-down solvent exchange-solvite-hydration strategy 174. This unique property of Janus hydrogels could potentially be applied to ocular adhesive hydrogels, particularly for intraocular retinopathy, paving the way for future advancements in this field.

Injectable hydrogels are polymer systems with good fluidity that, upon reaching the target site, undergo gelation triggered by factors such as self-crosslinking, temperature, pH, or light, enabling stable adhesion at the intended site. This distinct property makes injectable hydrogels especially well-suited for treating nasolacrimal duct disorders, minimally invasive glaucoma therapies, and vitreoretinal diseases. Although there has been significant research on injectable adhesive hydrogels for ocular applications, their use in treating diseases of ocular adnexa remains relatively limited 100, 147, 171. For example, future designs could involve adhesive hydrogels that can be injected through the punctum to seal the lacrimal duct opening, potentially serving as punctal plugs. Furthermore, these hydrogels could be engineered to carry drugs, exosomes, or other therapeutic agents for controlled release, offering both structural and therapeutic benefits.

The ocular surface microenvironment is intricately intertwined with the external milieu, and its microbiota plays a crucial role in maintaining microenvironmental homeostasis. When this microbiota is thrown into imbalance, the microenvironment is disrupted, potentially resulting in delayed wound healing and even exacerbating ocular infections. To address this, incorporating antimicrobial substances into material design is a viable strategy. Regarding ocular adhesive polymer hydrogel formulations, the majority are currently in the form of liquid drops or flexible solid tablets. However, the future may witness the emergence of ocular spray hydrogels. Compared to *in situ* hydrogels forming liquid drops, sprayed hydrogels offer a broader coverage area, swiftly forming a lightweight coating to minimize intraocular foreign body sensation. Moreover, the spray method significantly reduces bacterial contamination during application 175-177. Three-dimensionally printed hydrogels represent a rapid and efficient physical production method, allowing for continuous trial-and-error adjustments, and are currently a research hotspot 178, 179. Given that the shape of the eyeball and the curvature of the cornea vary among individuals, artificial intelligence could potentially recognize the unique shape of the ocular surface and cornea. By combining this technology with 3D printing, hydrogels that are more compatible with tissue trauma could be tailored specifically for each patient.

Regarding the biological properties of ocular adhesive hydrogels, there is a dearth of drug-device combination studies that seamlessly integrate the dual aspects of drug delivery and adhesive repair 180, 181. Such studies have the potential to augment repair mechanisms by adhering to wounds, encapsulating, and delivering drugs or bioactive substances for slow-release and controlled-release effects.

In summary, the eye is indispensable for vision, and while ocular adhesive hydrogels have garnered significant attention in recent research, numerous challenges persist due to the unique features of the eye, as well as the intricacies and variability of adhesive systems. Nonetheless, the application of adhesive polymer hydrogels in ophthalmology remains highly promising. To this end, we ought to devise multifunctional adhesive hydrogels tailored to specific ocular tissues and diseases, aiming to facilitate the diagnosis and treatment of ocular disorders, ultimately contributing to the advancement of human physical and mental well-being.

## Figures and Tables

**Table 1:** Adhesive hydrogels for ocular anterior section applications.

Application	Adherent tissue	Materials	Adhesion mechanism	Clinical research process	Ref.
Dry Eye	Cornea	MPEP(POSS-PEG-PPG)	Hydrogen bonds, hydrophobicity, mechanical interlocking	Pre-clinic	51
	Contact lens	PEG	Wet adhesive	Approved	52
	Cornea	AF127, Cu^2^-x Se NPs	Ionic bonds, covalent bonds (Schiff base)	Pre-clinic	53
	Lacrimal duct	CMC	Mechanical interlocking, cohesion	Pre-clinic	55
	Cornea	PBA, CMC, GSH	Hydrogen bonds, covalent bonds	Pre-clinic	60
Eye surface infection	Cornea	PAM, QCS, TA	Hydrogen bonds, electrostatic force, π-π stacking	Pre-clinic	68
	Cornea	F127, PEG-PPG-PEG, PLGA-PEI-PEG	Cohesion, mechanical interlocking, hydrogen bonds	Pre-clinic	69
	Cornea	FNPs, ISG	Cohesion, ionic bonds, hydrogen bonds	Pre-clinic	70
	Cornea	Gel	Mechanical interlocking	Pre-clinic	71
	Cornea	GAMA, contact lenses (with carboxylic acid)	Covalent bonds	Pre-clinic	72
Corneal wound surgery	Cornea	DPC-ECM	/	Approved	78
	Cornea	DPC- ECM, alginate, DOPA	Covalent bonds, ionic bonds, crosslinked structure	Pre-clinic	79
	Cornea	HA-cyclodextrin, HA-adamantane	Covalent bonds, cross-linked structures	Pre-clinic	80
	Cornea	Fibrin	Fibrinogen polymerization	Approved	85
	Cornea	PEG	Cohesion, hydrogen bonds, mechanical interlocking	Approved	20,86-88
	Cornea	4-arm-PEG-NHS, lysozyme	Covalent bonds, mechanical interlocking	Pre-clinic	89
	Cornea	Gel, TEA, N-VC	Hydrogen bonds, mechanical interlocking	Pre-clinic	93
	Cornea	GelMA, HAGM, PEGDA	Hydrogen bonds, covalent bonds, cross-linked structures, mechanical interlocking	Pre-clinic	94
	Cornea	Collagen I, F127DA, AF127,GelMA	Cross-linked structures, covalent bonds (Schiff base), hydrogen bonds, π-π stacking	Pre-clinic	95
	Cornea	GelMA, Odex	Dual network structure, covalent bonds (Schiff base), hydrogen bonds	Pre-clinic	96
	Cornea	GelMA, HA-NB	Wet adhesive, hydrogen bonds	Pre-clinic	97
	Cornea	Gel, DMA	Hydrogen bonds, covalent bonds	Pre-clinic	98
	Cornea	Gel, PEG, bovine stromal corneal extracellular matrix	Covalent bonding, double network structure	Pre-clinic	99
	Cornea	F127DA, HADA, DOPA	Wet adhesive, hydrogen bonds, crosslinked structure	Pre-clinic	100
	Cornea	AlgMA, ODex, dendritic polymers	Covalent bonding, inion bonds, triple-crosslinked double-network	Pre-clinic	101
Refractive surgery wounds	Cornea	PEG	Cohesion, hydrogen bonds, mechanical interlocking effects	Approved	105-107
Conjunctival defect	Conjunctiva	Acrylic resins	Hydrogen bonds	Pre-clinic	110
	Conjunctiva	PEG	Cohesion, hydrogen bonds, mechanical interlocking	Pre-clinic	113
	Conjunctiva	GelMA, OHA	Semi-interpenetrating polymer network, crosslinked structure, covalent bonds	Pre-clinic	114
	Conjunctiva, cornea	PEGDA, Alg-NHS, TA, Fe^3+^	Hydrogen bonds, covalent bonds, inion bonds	Pre-clinic	116

**Table 2:** Adhesive hydrogels for ocular posterior segment applications.

Application	Adherent tissue	Materials	Adhesion mechanism	Clinical research process	Ref.
Cataract	Cornea	Pluronic® F127	Hydrogen bonds, cohesion	Approved	125
	Anterior chamber	GenNLC, F127, F68	Hydrogen bonds, cohesion	Pre-clinic	128
Glaucoma	Cornea	PPI	Hydrogen bonds, covalent bonds	Pre-clinic	138
	Cornea	Thermosensitive elastin, silk elastin-like recombinants	Cohesion	Pre-clinic	139
	Cornea, conjunctiva	PAMAM, PLGA	Covalent bonds	Pre-clinic	140
	sclera	AOAQ, DMAA	Crosslinked structure, covalent bonds, hydrogen bonds	Pre-clinic	142
	Suprachoroidal space	HA-SH, PEGDA	Covalent bonds, hydrogen bonds, crosslinked structure	Pre-clinic	143
Vitreoretinopathy	Retina	PDA, GelCA, Cur	π-π stacking, hydrogen bonds	Pre-clinic	147
	Vitreous humor	PNaAMPS, PDMAAm	Hydrogen bonds, mechanical interlocking	Pre-clinic	148
	Retina	CS, Odex	Covalent bonds (Schiff base)	Pre-clinic	153
	Retina	Gel, HA, PDA	Wet adhesive	Pre-clinic	157
	Retina	Fibrin glue	Mechanical interlocking, ionic bonds	Pre-clinic	163
	Retina	PEG	/	Approved	[164.165]
	Retina	HA	Hydrogen bonds, ionic bonds, mechanical interlocking	Approved	166
Open eye trauma	Cornea, conjunctiva, sclera	N-isopropylacrylamide, butyl acrylate	Mechanical interlocking, diffusion, hydrogen bonds, covalent bonds	Pre-clinic	171
	Cornea	PEG, CV	Hydrogen bonds, mechanical interlocking	Pre-clinic	172
	Cornea	PDA	Cohesion, covalent bonds	Pre-clinic	173
